# Primary prophylaxis of invasive fungal infections in patients with haematological malignancies: 2017 update of the recommendations of the Infectious Diseases Working Party (AGIHO) of the German Society for Haematology and Medical Oncology (DGHO)

**DOI:** 10.1007/s00277-017-3196-2

**Published:** 2017-12-07

**Authors:** Sibylle C. Mellinghoff, Jens Panse, Nael Alakel, Gerhard Behre, Dieter Buchheidt, Maximilian Christopeit, Justin Hasenkamp, Michael Kiehl, Michael Koldehoff, Stefan W. Krause, Nicola Lehners, Marie von Lilienfeld-Toal, Annika Y. Löhnert, Georg Maschmeyer, Daniel Teschner, Andrew J. Ullmann, Olaf Penack, Markus Ruhnke, Karin Mayer, Helmut Ostermann, Hans-H. Wolf, Oliver A. Cornely

**Affiliations:** 1 Cologne Excellence Cluster on Cellular Stress Responses in Aging-Associated Diseases (CECAD), University of Cologne, Cologne, Germany; 2 Department I of Internal Medicine, German Centre for Infection Research (DZIF), University Hospital of Cologne, University of Cologne, Cologne, Germany; 3 Department of Oncology, Haematology, Haemostaseology and Stem Cell Transplantation, University Hospital RWTH Aachen, Aachen, Germany; 4 Department I of Internal Medicine, Haematology and Oncology, University Hospital Dresden, Dresden, Germany; 5 Division of Haematology and Oncology, Leipzig University Hospital, Leipzig, Germany; 6 Department of Internal Medicine–Haematology and Oncology, Mannheim University Hospital, Heidelberg University, Mannheim, Germany; 7 Department of Stem Cell Transplantation, University Medical Centre Hamburg-Eppendorf, Hamburg, Germany; 8 Clinic for Haematology and Medical Oncology with Department for Stem Cell Transplantation, University Medicine Göttingen, Göttingen, Germany; 9 Department I for Internal Medicine, Klinikum Frankfurt (Oder), Frankfurt (Oder), Germany; 10 Department of Bone Marrow Transplantation, West German Cancer Centre, University Hospital of Essen, University of Duisburg-Essen, Duisburg, Germany; 11 Department V for Internal Medicine, University Hospital Erlangen, Erlangen, Germany; 12 Department of Internal Medicine V, Heidelberg University Hospital, Heidelberg, Germany; 13 Department of Haematology and Oncology, University Hospital of Jena, Jena, Germany; 14 Department of Haematology, Oncology and Palliative Care, Klinikum Ernst von Bergmann, Potsdam, Germany; 15 Department of Haematology, Medical Oncology, and Pneumology, University Medical Center of the Johannes Gutenberg University Mainz, Mainz, Germany; 16 Department II of Internal Medicine, University Hospital Wuerzburg, Wuerzburg, Germany; 17 Department for Haematology, Oncology and Tumour immunology, Charité Universitätsmedizin Berlin, Berlin, Germany; 18 Department of Haematology and Oncology, Paracelsus-Kliniken Osnabrück, Osnabrück, Germany; 19 Department III of Internal Medicine, University Hospital Bonn, Bonn, Germany; 20 Department of Haematology and Oncology, University of Munich, Munich, Germany; 21 Department IV of Internal Medicine, University Hospital Halle, Halle, Germany; 22 Clinical Trials Centre Cologne (ZKS Köln), University of Cologne, Cologne, Germany

**Keywords:** Invasive fungal infection, Antifungal prophylaxis, Itraconazole, Fluconazole, Posaconazole, Amphotericin B, Liposomal, Isavuconazole

## Abstract

Immunocompromised patients are at high risk of invasive fungal infections (IFI), in particular those with haematological malignancies undergoing remission-induction chemotherapy for acute myeloid leukaemia (AML) or myelodysplastic syndrome (MDS) and recipients of allogeneic haematopoietic stem cell transplants (HSCT). Despite the development of new treatment options in the past decades, IFI remains a concern due to substantial morbidity and mortality in these patient populations. In addition, the increasing use of new immune modulating drugs in cancer therapy has opened an entirely new spectrum of at risk periods. Since the last edition of antifungal prophylaxis recommendations of the German Society for Haematology and Medical Oncology in 2014, seven clinical trials regarding antifungal prophylaxis in patients with haematological malignancies have been published, comprising 1227 patients. This update assesses the impact of this additional evidence and effective revisions. Our key recommendations are the following: prophylaxis should be performed with posaconazole delayed release tablets during remission induction chemotherapy for AML and MDS (AI). Posaconazole iv can be used when the oral route is contraindicated or not feasible. Intravenous liposomal amphotericin B did not significantly decrease IFI rates in acute lymphoblastic leukaemia (ALL) patients during induction chemotherapy, and there is poor evidence to recommend it for prophylaxis in these patients (CI). Despite substantial risk of IFI, we cannot provide a stronger recommendation for these patients. There is poor evidence regarding voriconazole prophylaxis in patients with neutropenia (CII). Therapeutic drug monitoring TDM should be performed within 2 to 5 days of initiating voriconazole prophylaxis and should be repeated in case of suspicious adverse events or of dose changes of interacting drugs (BIItu). General TDM during posaconazole prophylaxis is not recommended (CIItu), but may be helpful in cases of clinical failure such as breakthrough IFI for verification of compliance or absorption.

**Electronic supplementary material:**

The online version of this article (10.1007/s00277-017-3196-2) contains supplementary material, which is available to authorized users.

## Introduction

Invasive fungal infections (IFIs) cause substantial morbidity and mortality in patients with haematological malignancies, especially in those receiving remission-induction therapy for acute myeloid leukaemia (AML) or myelodysplastic syndrome (MDS) and allogeneic haematopoietic stem cell transplantation (HSCT) [53, 97]. In addition, the increasing use of new immune modulating drugs in cancer therapy has opened up an entirely new spectrum of patients at risk [57, 84]. Patients with haematological or oncological diseases without risk for prolonged neutropenia (< 500 cells/μL > 7 days) are not at increased risk for IFI and should therefore not receive routine prophylaxis (DI).

Epidemiology of IFI varies upon host and environmental factors [63]. *Aspergillus* spp. and *Candida* spp. cause most cases of IFI in haematological patients [65, 81]. However, the introduction of routine prophylaxis for patients at high risk for IFI plus local environmental factors have caused a shift in epidemiology, in particular to non-albicans *Candida* spp. such as *C. glabrata*, *C. krusei* and *C. Tropicalis* [28, 78, 99, 112]. Despite improvements in diagnosis and treatment, IFI-associated mortality remains high [54, 78], and thus, antifungal prophylaxis represents an important strategy in patients at high risk for IFI.

Since the 2014 edition of these recommendations [95], seven clinical trials regarding antifungal prophylaxis in patients with haematological malignancies have been published, comprising 1227 patients. This 2017 update intends to facilitate evidence-based decision making in daily clinical practice. Additional evidence from clinical trials and its impact on changes compared to our previous recommendations will be discussed.

## Design and methods

The guideline was prepared by German clinical experts in haematology, oncology, stem cell transplantation and infectious diseases in a stepwise consensus process. Systematic literature search was conducted by OAC and SCM as previously described [15, 95]. Data were extracted and tabulated; preliminary recommendations for each patient group were proposed for discussion and sent to the committee, i.e. all authors. Tables were revised after email-based discussion and put up for final discussion at a telephone conference on June 20th, 2017. If no unanimous consensus was reached, majority vote of the conference was adopted. The final version of this guideline was approved by the AGIHO plenary session on September 30th, 2017.

A major change to the 2014 edition of this guideline is the elimination of the recommendations for allogeneic HSCT recipients in order to avoid duplication. Instead, we refer to the guidelines for infectious complications after allogeneic HSCT provided by the Infectious Diseases Working Party of the German Society for Haematology and Medical Oncology [104] and specifically developed for this patient group. For prophylaxis of *Pneumocystis jirovecii* pneumonia, please refer to the guidelines for primary prophylaxis of bacterial infections and *Pneumocystis jirovecii* pneumonia in patients with haematological malignancies and solid tumours provided by the AGIHO [75].

In contrast to the last edition, we used grading for strength of recommendation and quality of evidence (Table 1) established by the European Society for Clinical Microbiology and Infectious Diseases (ESCMID) and the European Confederation of Medical Mycology (ECMM) [17]. When propositions did not change since 2014, the reader may refer to that previous publication [95]. The synopsis of our recommendations is given in Tables 2, 3 and 4.

**Table 1:** ESCMID-ECMM Grading 2017

Category, grade		Definition
Strength of recommendation	A	Strongly supports a recommendation for use
	B	Moderate evidence to support a recommendation for use
	C	Poor evidence to support a recommendation
	D	Supports a recommendation against use
Quality of evidence—level	I	Evidence from ≥ 1 properly randomised controlled trial
	II	Evidence from ≥ 1 well-designed clinical trial, without randomisation; from cohort or case-controlled analytic studies (preferably from > 1 centre); from multiple time series; or from dramatic results from uncontrolled experiments
	III	Evidence from opinions of respected authorities, based on clinical experience, descriptive studies or reports of expert committees
Quality of evidence—index (for level II)	r	Meta-analysis or systematic review of randomised controlled trials
	t	Transferred evidence, that is, results from different patients’ cohorts, or similar immune-status situation
	h	Comparator group is a historical control
	u	Uncontrolled trial
	a	Published abstract (presented at an international symposium or meeting)

**Table 2:** Recommended antifungal prophylaxis in patients with neutropenia (< 500 cells/μL > 7 days)

Intention	Intervention	SoR	QoE
Prevent IFI in patients with neutropenia (< 500 cells/μL > 7 days), excluding alloSCT^a^	Posaconazole	A	I^b^
		B	III^c^
	Amphotericin B, liposomal, inhalation	B	II^d^
	Amphotericin B, liposomal, iv	C	I
	Caspofungin	C	I
	Fluconazole	C	I
	Itraconazole	C	I
	Itraconazole, iv	C	I
	Voriconazole	C	II
	Amphotericin B deoxycholate	D	I
	Micafungin	C	IIh
	Isavuconazole	C	IIu

^a^Currently, no recommendations for ALL patients applicable

^b^Strong recommendation in AML/MDS remission induction chemotherapy only

^c^Other settings, e.g. very severe aplastic anaemia and palliative treatment of MDS

^d^All patients received fluconazole—dose and route were not reported

**Table 3:** Dosage of recommended drugs (please refer to Table 2)

Drug	Dosage
Posaconazole, oral suspension	200 mg tid po
Posaconazole, tablet	300 mg qd po (bid on day 1)
Posaconazole, iv	300 mg/day iv (bid on day 1)
Amphotericin B, liposomal, inhalation	12.5 mg biw
Amphotericin B, liposomal, iv	50 mg q 48 h or 5 mg/kg biw (CI) 15 mg/kg single infusion (CIII)
Caspofungin	50 mg qd iv
Fluconazole	400 mg qd po
Itraconazole, capsules	Any dose
Itraconazole, oral solution	2.5–7.5 mg/kg/day or 200 mg
Itraconazole, iv	200 mg qd iv
Voriconazole	200 mg bid iv
Amphotericin B deoxycholate	Any dose
Micafungin	50 mg iv
Isavuconazole	200 mg/d iv (tid on days 1–2)

**Table 4:** Recommendations on therapeutic drug monitoring during antifungal prophylaxis

Intention	Intervention		SoR	QoE
	Drug	Target level		
Achieve exposure effective for antifungal prophylaxis and reduce toxicity	Voriconazole	1–2 mg/L	B	IItu
	Posaconazole	> 500 ng/ml	C	IItu

In order to provide a complete overview, this paper includes tables of the trials on antifungal prophylaxis published to date by compound and comprising information about author, publishing year, trial design, medication/daily dose per treatment group, number of patients, risk factors, percentage of proven, probable and possible IFI and attributable and overall mortality (Supplementary Tables 5 to 11, updating previous information published here[15, 95]). Two authors (SCM and AYL) double-checked the detailed information provided.

Recommendations apply for adult patients only, and clinical trials evaluating antifungal prophylaxis exclusively in paediatric patients are beyond the scope of our review. Status of approval of drugs in national health care systems was not taken into account.

These recommendations are evidence-based, but not necessarily follow approved indications or the respective labelling of antifungal compounds as they may differ substantially between countries and over time.

## Results

### Triazoles

Triazoles represent an important class of antifungal drugs for both prevention and treatment of *Aspergillus* spp. and certain yeasts including many *Candida* spp. However, *A. fumigatus* being resistant to triazoles has emerged within the past decade [9, 45]. The SEPIA study assessed the epidemiology of invasive aspergillosis (IA) and azole resistant *Aspergillus* spp. in patients with acute leukaemia in 19 haematology centres in Germany. The authors found resistance in two in 179 (1.1%) cases [53]. A European expert group recently published a statement proposing that local resistance rates of < 5% should not trigger changes in national or international management recommendations [108]. Therefore, a modification of antifungal prophylaxis in Germany does not appear to be warranted. The 5% cut-off was not reached in any of the SEPIA study sites [53].

#### Fluconazole

Since 2014, one prospective study on fluconazole prophylaxis was conducted. This small prospective study compared posaconazole with fluconazole for prophylaxis in 37 AML patients during induction and consolidation chemotherapy. IFI rates did not differ significantly (10 and 7 cases), but posaconazole direct costs exceeded fluconazole considerably (24€ and 2400€, respectively) [6]. However, posaconazole was demonstrated to have a survival benefit in prospective randomised controlled clinical trials (RCTs) when given for fungal prophylaxis treatment and decreased indirect and overall costs [22, 86]. Fluconazole is a weaker CYP3A4 inhibitor than other azoles and, specifically, fluconazole prophylaxis has been used in acute lymphoblastic leukaemia (ALL) induction chemotherapy, but there are no reliable data to support a recommendation for prophylaxis in this setting. Thus, our recommendation (CI) regarding fluconazole prophylaxis in patients with neutropenia remains unchanged.

#### Itraconazole

A non-comparative prospective trial evaluated the administration of itraconazole prophylaxis in AML patients [52]. Eighty-four patients received 200-mg oral solution twice daily during induction, re-induction and consolidation chemotherapy. IFI occurred in 3.4%, adverse events occurred in 7%, none leading to discontinuation. The study added only little more information to the already vast body of evidence on the prophylactic use of the itraconazole oral solution; therefore, the recommendation with poor evidence to support the prophylactic use of itraconazole (CI) did not change.

#### Isavuconazole

Isavuconazole is a novel antifungal approved in 2015. An open-label dose escalation study in 23 patients with AML was conducted (ClinicalTrials.gov identifier NCT00413439): 11 patients received 200 mg and 12 received 400 mg isavuconazole intravenously as antifungal prophylaxis [16]. Two patients developed possible IFI; most adverse events were mild or moderate, leading to discontinuation in four cases. In this small, phase II study, isavuconazole appeared safe and tolerable as prophylaxis in immunosuppressed high-risk patients. Only a well-designed RCT could provide solid evidence of prophylactic efficacy of isavuconazole. Currently, there is poor evidence to recommend prophylactic isavuconazole (CIIu).

#### Posaconazole

We strongly recommend antifungal prophylaxis with posaconazole (AI) in patients with neutropenia. Posaconazole is available in different formulations: oral suspension, tablet and iv formulation. Strong recommendation for posaconazole use bases on a large RCT with the oral suspension [22].

Posaconazole oral suspension was used in a single-centre, retrospective cohort study since 2014. This study compared clinical effectiveness of posaconazole with fluconazole in 130 patients receiving prophylaxis during first induction or first re-induction chemotherapy for AML or MDS. The primary endpoint was possible, probable or definite breakthrough IFI. Efficacy of posaconazole was superior to fluconazole, probable/definite breakthrough IFI occurred in 9.2 and 27.0%, respectively. High differences of IFI in different studies can be explained by incomplete data sets due to retrospective acquisition and varying definitions of IFI despite wide spread use of EORCT criteria. Additionally, centre effects driven by differences in rigour of diagnostic strategies may contribute to discrepancy. These results support our previous strong recommendation for antifungal prophylaxis with posaconazole in patients at high risk for IFI (AI). In 2015, a retrospective analysis of 70 AML patients after induction chemotherapy found the frequent necessity of systemic antifungal treatment for probable or proven IFI despite prophylaxis with posaconazole oral suspension [92].

No data are available for populations with persisting neutropenia, e.g. very severe aplastic anaemia or MDS treated with hypomethylating agents. Thus, one must extrapolate from findings of other high-risk neutropenic patient studies [22]. The group recommends posaconazole prophylaxis in such clinical settings (BIIt), unless such prophylaxis is contraindicated because of drug-drug interactions, e.g. ALL patients [5, 20].

No evidence-based recommendations can be made on the duration of prophylaxis in patients with persisting neutropenia. There is poor evidence regarding posaconazole prophylaxis during AML consolidation therapy (CIIt). Further prospective trials on antifungal prophylaxis in patients with ALL, aplastic anaemia or MDS are required to give evidence-based recommendations in future.

Posaconazole oral suspension has limited bioavailability underlining the need for better absorbable formulations [24, 92]. Since 2014, two studies have been published on prophylaxis with posaconazole delayed release (DR) tablets. One study compared 200 and 300 mg tablets in 54 patients to evaluate pharmacokinetics and safety profile (NCT01777763). The exposure target of a steady-state average concentration of > 500 ng/mL was reached in 15 of 19 patients receiving 200 mg once daily and in 31 of 32 on 300 mg once daily. Tablets were well tolerated [27]. The second study characterised posaconazole tablet pharmacokinetic and safety in 210 patients with neutropenia following chemotherapy for haematological malignancy or recipients of allogeneic HSCT. Patients took posaconazole 300 mg DR tablets once daily independent of food intake. Pre-specified exposure targets were achieved in almost all patients. The drug was well tolerated and safe, similar to posaconazole oral suspension [18]. The tablet formulation of posaconazole is safe, effective and provides predictable absorption. We thus recommend posaconazole tablets as drug of choice for IFI prevention in AML and MDS patients (AI).

One study evaluated pharmacokinetics and safety of intravenous posaconazole in antifungal prophylaxis of neutropenic patients with AML, MDS or in the context of allogeneic HSCT [20]. In total, 237 received 300 mg posaconazole iv twice daily on day 1 and thereafter 300 mg iv once daily for up to 28 days. Average concentrations were reached in 94% of patients between 500 and 2500 ng/mL. The most common treatment-related adverse events were diarrhoea, nausea and rash (8, 5 and 5%, respectively). We recommend posaconazole iv in patients when oral formulations are not appropriate, but emphasise the need for further randomised trials.

#### Voriconazole

Voriconazole has been shown to be efficacious in the treatment of IA [44], but—apart from haematopoietic stem cell recipients—data on prophylaxis in patients at risk for IFI is still scarce [62, 111]. One retrospective study compared safety and efficacy of voriconazole and oral suspension posaconazole prophylaxis in patients with haematological malignancies (*n* = 200) [39]. IFI occurred in 0 and 3%, respectively; symptomatic adverse events were more frequent in the voriconazole group (6 and 0%). Our CII recommendation for voriconazole remains therefore unchanged.

#### Clotrimazole, miconazole and ketoconazole

No additional literature has been published since 2014. There is poor evidence to support the prophylactic use of clotrimazole, miconazole or ketoconazole (DII).

#### Echinocandins

Resistance to drugs of the echinocandin class remains low for most *Candida* spp., e.g. *C. albicans* at < 3% [10]. In contrast, *C. glabrata* shows increasing in vitro resistance to this drug class [1, 80]. Expanding use of echinocandins for prophylaxis in patients with high risk of invasive candidiasis has some potential to contribute to emergence of resistance. We hence recommend to consider antifungal prophylaxis with echinocandins on the basis of local epidemiology (BIII) [35].

#### Anidulafungin and caspofungin

No additional relevant data have been published since 2014; our recommendation in patients with neutropenia remains unchanged (CI).

#### Micafungin

No prospective clinical trial on micafungin prophylaxis in the non-transplant setting was published since 2014. Results from a retrospective single-centre observational study comparing micafungin 50 mg iv with posaconazole 200 mg orally (historical control) in the prevention of IFI in neutropenic patients with haematological malignancies (*n* = 302) showed that there was no statistically significant difference in IFI rates (6.0 versus 5.4%) [73]. The authors propose micafungin as a good alternative for antifungal prophylaxis in patients with neutropenia while posaconazole and liposomal amphotericin B should remain first-line therapy. Our recommendation in patients with neutropenia remains unchanged (CIIh).

#### Polyenes

##### Liposomal amphotericin B

Prophylactic aerosolized liposomal amphotericin B in severely neutropenic patients significantly reduced invasive pulmonary aspergillosis (IPA) rates, resulting in a BII recommendation in 2014 [87]. A recently published cohort study confirmed that prophylactic liposomal amphotericin B inhalation resulted in a substantial decrease in IPA incidence [12]; 235 AML patients inhaled 12.5 mg twice weekly from initiation of remission induction chemotherapy until recovery of their neutrophils. The primary endpoint was incidence of proven or probable IPA until 28 days after neutrophil recovery. IPA rates were 9.5% in the liposomal amphotericin B and 23.4% in the historical no-prophylaxis control group. Liposomal amphotericin B inhalation appeared cost saving. Considering increasing azole resistance, non-azoles may become an option for future strategies in antifungal prophylaxis. Our recommendation to BII recommendation remains unchanged.

A prospective non-comparative trial has demonstrated feasibility and safety of prophylaxis with a single 15-mg/kg intravenous L-AmB dose in 48 AML patients undergoing induction chemotherapy [2]. Apart from six patients with hypokalaemia, no grade 3–4 adverse events were reported. This approach needs validation by further clinical trials and can only be recommended with poor evidence at this time (CIIu).

Liposomal amphotericin B prophylaxis was also evaluated in adult patients with ALL receiving remission-induction chemotherapy (NCT01259713). Currently, there is no approved standard of care for this group of patients regarding antifungal prophylaxis. Azole antifungal drugs are problematic because of drug-drug interactions with vin-caalkaloids, an integral component of ALL induction chemotherapy regimens [5, 19]. The authors hypothesised that liposomal amphotericin B is an alternative due to its broad spectrum of activity [14, 21, 56]. Yet, IFI rates in the liposomal amphotericin B group (7.9%) did not significantly differ from the placebo group (11.7%). Given the high IFI rate in the placebo group, further clinical trials are needed to define an adequate antifungal prophylaxis strategy in ALL patients during remission-induction. Until then, there is poor evidence to recommend intravenous liposomal amphotericin B for prophylaxis in ALL (CI).

Amphotericin B deoxycholate has been shown to be too toxic and therefore is not recommended for prophylactic use (DI).

#### Nystatin

The use of nystatin mouthwash was compared to placebo in patients with haematological malignancies (*n* = 158) and was—surprisingly—found effective for prophylaxis of pulmonary IFI (IFI rates 1.6 and 27.7%, respectively) [46]. Due to the missing mechanistic explanation of the result and the uncertain attribution of colonisation and IFI, the study did not impact our recommendations on antifungal prophylaxis in neutropenic patients (DII).

### Risk factors for IFI

#### Novel targeted cancer therapy

New drug classes for haematological and oncologic diseases such as tyrosine kinase inhibitors (TKI) and other immunomodulatory drugs put a broader spectrum of patients at risk for IFI [84].

Among TKI, in particular inhibitors of bruton tyrosine kinase (BTK) [77, 84], mammalian target of rapamycin (mTOR) [31, 72, 88], janus kinase (JAK) [41, 70, 113] and phosphatidylinositol 3 kinase (PI3K) delta [58] showed attributable increase of risk of IFI. Targeting critical components of the immune system, they impair diverse features of immune cells (e.g. dendritic cells, T cells) [42, 91, 114]. However, underlying haematological disease, recent treatment, as well as neutropenia put these patients at an increased baseline risk for IFI. Currently, it remains unclear, if antifungal prophylaxis is indicated in these cases.

Inhibition of immune checkpoints, e.g. programmed cell death protein 1 (PD1) or cytotoxic T lymphocyte-associated protein 4 (CTLA4), shows wide-ranging, mostly immune-related adverse events [7, 59, 98]. Subsequent immunosuppression, primarily including corticosteroids, may result in opportunistic infections including fungi [32, 57, 110]. Prospective clinical trials may help optimizing management of immune-related adverse events.

Hypomethylating agents such as azacitidine put patients with AML or MDS at risk of IFI (probable/proven IFI 1.6%, *n* = 121, to 8.3%, *n* = 64) [30, 82]. Further, independent risk factors are low neutrophil and platelet counts [68], as well as prior intensive chemotherapy [30, 68]. Evaluation of risk factors should precede prescription of hypomethylating agents and antifungal prophylaxis could be considered accordingly.

Targeting CD20 leads to prolonged B cell depletion and in rare cases to late-onset neutropenia [29, 106, 109]. One retrospective case-control study reported that a significantly higher IFI rate was reported in patients treated with rituximab regimens compared to chemotherapy alone (41.7 vs. 17.1% among all infections, *n* = 69) [61]. Large randomised trials evaluating efficacy and safety of adding rituximab to standard chemotherapy did not find increased IFI rates [13, 38]. Antifungal prophylaxis should only be considered in case of additional risk factors.

Further antibodies target CD19, CD33 or interleukin-2 (IL-2). Low evidence on risk of IFI makes it difficult to give specific recommendations and guidelines on empiric or pre-emptive therapy should be followed [43, 74]. In rare occasions where CD52 antibody is part of the antineoplastic strategy, mould directed prophylaxis should be considered [51, 71]. Bispecific antibodies frequently cause neutropenic fever and infections, but direct causal relationship with these drugs is difficult to attribute being used in patients with advanced lymphoma at high risk for infection anyhow [85]. An increased risk for IFI has not been reported to date.

Given the high attributable mortality of IFI, the individual risk of patients treated with the drug classes above should be evaluated, and antifungal prophylaxis prescribed on case by case basis. Guidance that is more precise needs prospective trials focussed on infections.

#### Infection control for prevention of IFI

Infection-control measures in the haematological and oncologic setting are heterogeneous and contentious, particularly about transmission of fungi. The *Robert Koch-Institute* in Germany published recommendations on hygiene requirements for the medical care of immunocompromised patients [3]. However, most recommendations are based on expert opinion rather than actual published evidence. We reviewed recent trials on infection-control measures intending to prevent or reduce the rate of IFI.

Most studies focusing on the role of protective isolation are non-randomised and biased by renovation and reconstruction [67]. Available studies suggested clinical benefit of air filters and positive pressure environments, but mainly evaluated fungal conidia air concentration instead of patient outcome [55, 76]. None was randomised. One meta-analysis confirmed the low level of available evidence. No data showing a reduction of mould infections are available [90].

Surgical masks are used for protection of immunocompromised high-risk patients, but a clinical benefit has not been demonstrated [64]. One RCT compared 80 adult patients treated for acute leukaemia or HSCT regarding standard hospital hygiene procedures with or without wearing masks. A reduction of IFI was not seen (proven/probable IFI in 19.5 and 20.5%) [66]. In contrast, one study compared neutropenic patients wearing surgical masks during hospital construction with a historical control and found a reduction in *Aspergillus* spp. infections [83]. Specific settings may justify the use of well-fitting face masks; routine use seems inappropriate.

The value of germ-reduced diet including so-called “neutropenic diet” is unproven. No RCT proved a benefit for prevention of infection and related outcomes. All studies had limitations regarding confounding interventions, outcome definitions, intervention and control diets [25, 33, 69, 105, 107].

Further clinical implications include appropriate hand hygiene. This aspect has recently been pointed out within the context of *Candida auris* transmission considering that hands can be key vectors in the transmission of yeasts [89]. Housing of patients as well as limitation of environmental exposure to air-borne conidia are matters of infection control and may outweigh impact of chemoprophylaxis. Because of difficulties in randomisation evidence remains low.

Due to lack of evidence, we do not provide recommendations for clinical practice.

### Role of therapeutic drug monitoring

Therapeutic drug monitoring (TDM) of serum samples may improve efficacy and safety of antifungal prophylaxis. Two variables influence the potential utility of TDM: variable pharmacokinetics and a clear correlation between plasma drug concentration and efficacy or toxicity [47, 94].

Voriconazole meets both criteria: pharmacokinetics are variable while exhibiting difficulty to predict drug dose-exposure relationship [8, 50, 100, 101], and serum concentration was linked to efficacy [79, 102] and toxicity [26, 48, 79, 96, 100, 102, 115]. Voriconazole serum levels were often out of target range at the initiation of antifungal prophylaxis, 18% were sub-therapeutic (< 1 mg/L) and 11% too high (> 5.5 mg/L) (*n* = 107) [37]. Based on current literature, we recommend a concentration of 1–2 mg/L for prophylactic efficacy [79, 102]. In addition, concentrations > 5–6 mg/L should be avoided to prevent central nervous system and liver toxicity [26, 79, 102, 115]. TDM should be done within 2 to 5 days of treatment initiation, and repeatedly in case of suspicious adverse events, or initiation or termination of interacting drugs (BIItu).

There is no well-defined minimum serum concentration for posaconazole prophylaxis. Neither did serum concentrations of posaconazole correlate with efficacy nor with toxicity in the two large RCT [22, 103]. A level of 500 mg/L has been proposed, but was merely extrapolated from itraconazole data [34]. Despite lack of evidence [23, 49] to support a specific reference range, there is a general consensus of 500 to 700 mg/L being a desirable lower bound [4, 11, 40, 60, 93]. A retrospective study analysing posaconazole serum levels of 31 patients described lower serum levels in 43% of patients associated with advanced age and mucositis [36]. Routine TDM is not recommended (CIItu). Yet, in cases of clinical failure such as breakthrough IFI, it may be helpful for verification of compliance or absorption. An important parameter influencing usefulness of TDM is the turnaround time from sampling to result.

## Conclusion

There is good evidence to recommend antifungal prophylaxis with posaconazole as oral suspension or—preferably—tablet in patients with remission induction chemotherapy for AML and MDS (AI). Posaconazole iv administration can be considered in those cases unable to take or absorb oral formulation. Liposomal amphotericin B did not significantly decrease IFI rates in ALL induction chemotherapy patients (CI). Since the IFI rate in ALL patients is considerable, further clinical trials are needed to find effective antifungal prophylaxis.

TDM should be performed within 2 to 5 days of voriconazole prophylaxis initiation and should be repeated in case of suspicious adverse events or dose changes of interacting drugs (BIItu). TDM is not generally necessary during posaconazole prophylaxis (CIItu), although in individual cases, for example potential breakthrough infection, it may be helpful to evaluate compliance, absorption and likelihood of IF.

Compared to the 2014 edition of this guideline, a further change is the elimination of the recommendations for allogeneic HSCT recipients regarding antifungal prophylaxis. We moved them to our guidelines specifically developed for this group of patients [104].

## Electronic supplementary material

ESM 1 (DOCX 232 kb)

## References

[1] Alexander BD, Johnson MD, Pfeiffer CD (2013). Increasing echinocandin resistance in Candida Glabrata: clinical failure correlates with presence of FKS mutations and elevated minimum inhibitory concentrations. Clin Infect Dis.

[2] Annino L, Chierichini A, Anaclerico B (2013). Prospective phase II single-center study of the safety of a single very high dose of liposomal amphotericin B for antifungal prophylaxis in patients with acute myeloid leukemia. Antimicrob Agents Chemother.

[3] Anonymous (2010). Requirements for hygiene in the medical care of immunocompromised patients. Recommendations from the Committee for Hospital Hygiene and Infection Prevention at the Robert Koch institute (RKI). Bundesgesundheitsblatt, Gesundheitsforschung, Gesundheitsschutz.

[4] Ashbee HR, Barnes RA, Johnson EM (2014). Therapeutic drug monitoring (TDM) of antifungal agents: guidelines from the British Society for Medical Mycology. J Antimicrob Chemother.

[5] Bassan R, Hoelzer D (2011). Modern therapy of acute lymphoblastic leukemia. J Clin Oncol.

[6] Bertz H, Drognitz K, Lubbert M (2014). No difference between posaconazole and fluconazole antifungal prophylaxis and mycological diagnostics except costs in patients undergoing AML chemotherapy: a 1-year "real-life" evaluation. Ann Hematol.

[7] Brahmer JR, Tykodi SS, Chow LQ (2012). Safety and activity of anti-PD-L1 antibody in patients with advanced cancer. N Engl J Med.

[8] Bruggemann RJ, Blijlevens NM, Burger DM (2010). Pharmacokinetics and safety of 14 days intravenous voriconazole in allogeneic haematopoietic stem cell transplant recipients. J Antimicrob Chemother.

[9] Camps SM, Van Der Linden JW, Li Y (2012). Rapid induction of multiple resistance mechanisms in aspergillus fumigatus during azole therapy: a case study and review of the literature. Antimicrob Agents Chemother.

[10] Castanheira M, Woosley LN, Diekema DJ (2010). Low prevalence of fks1 hot spot 1 mutations in a worldwide collection of Candida strains. Antimicrob Agents Chemother.

[11] Chau MM, Kong DC, Van Hal SJ (2014). Consensus guidelines for optimising antifungal drug delivery and monitoring to avoid toxicity and improve outcomes in patients with haematological malignancy, 2014. Intern Med J.

[12] Chong GL, Broekman F, Polinder S (2015). Aerosolised liposomal amphotericin B to prevent aspergillosis in acute myeloid leukaemia: efficacy and cost effectiveness in real-life. Int J Antimicrob Agents.

[13] Coiffier B, Lepage E, Brière J (2002). CHOP chemotherapy plus rituximab compared with CHOP alone in elderly patients with diffuse large-B-cell lymphoma. N Engl J Med.

[14] Cornely OA, Arikan-Akdagli S, Dannaoui E (2014). ESCMID† and ECMM‡ joint clinical guidelines for the diagnosis and management of mucormycosis 2013. Clin Microbiol Infect.

[15] Cornely OA, Bohme A, Buchheidt D (2009). Primary prophylaxis of invasive fungal infections in patients with hematologic malignancies. Recommendations of the Infectious Diseases Working Party of the German Society for Haematology and Oncology. Haematologica.

[16] Cornely OA, Bohme A, Schmitt-Hoffmann A (2015). Safety and pharmacokinetics of isavuconazole as antifungal prophylaxis in acute myeloid leukemia patients with neutropenia: results of a phase 2, dose escalation study. Antimicrob Agents Chemother.

[17] Cornely OA, Cuenca-Estrella M, Meis JF (2014). European Society of Clinical Microbiology and Infectious Diseases (ESCMID) fungal infection study group (EFISG) and European Confederation of Medical Mycology (ECMM) 2013 joint guidelines on diagnosis and management of rare and emerging fungal diseases. Clin Microbiol Infect.

[18] Cornely OA, Duarte RF, Haider S (2016). Phase 3 pharmacokinetics and safety study of a posaconazole tablet formulation in patients at risk for invasive fungal disease. J Antimicrob Chemother.

[19] CornelyOA, LeguayT, MaertensJet al. (2017) Randomized comparison of liposomal amphotericin B versus placebo to prevent invasive mycoses in acute lymphoblastic leukaemia. J Antimicrob Chemother 10.1093/jac/dkx133 PMC5890735 28575414

[20] Cornely OA, Leguay T, Maertens J (2017). Randomized comparison of liposomal amphotericin B versus placebo to prevent invasive mycoses in acute lymphoblastic leukaemia. J Antimicrob Chemother.

[21] Cornely OA, Maertens J, Bresnik M (2007). Liposomal amphotericin B as initial therapy for invasive mold infection: arandomized trial comparing a high–loading dose regimen with standard dosing (AmBiLoad trial). Clin Infect Dis.

[22] Cornely OA, Maertens J, Winston DJ (2007). Posaconazole vs. fluconazole or itraconazole prophylaxis in patients with neutropenia. N Engl J Med.

[23] Cornely OA, Ullmann AJ (2011). Lack of evidence for exposure-response relationship in the use of posaconazole as prophylaxis against invasive fungal infections. Clin Pharmacol Ther.

[24] Courtney R, Wexler D, Radwanski E (2004). Effect of food on the relative bioavailability of two oral formulations of posaconazole in healthy adults. Br J Clin Pharmacol.

[25] Demille D, Deming P, Lupinacci P (2006). The effect of the neutropenic diet in the outpatient setting: a pilot study. Oncol Nurs Forum.

[26] Denning DW, Ribaud P, Milpied N (2002). Efficacy and safety of voriconazole in the treatment of acute invasive aspergillosis. Clin Infect Dis.

[27] Duarte RF, Lopez-Jimenez J, Cornely OA (2014). Phase 1b study of new posaconazole tablet for prevention of invasive fungal infections in high-risk patients with neutropenia. Antimicrob Agents Chemother.

[28] Enoch DA, Yang H, Aliyu SH (2017). The changing epidemiology of invasive fungal infections. Methods Mol Biol (Clifton, N.J.).

[29] Faderl S, Thomas DA, O'brien S (2003). Experience with alemtuzumab plus rituximab in patients with relapsed and refractory lymphoid malignancies. Blood.

[30] Falantes JF, Calderon C, Marquez-Malaver FJ (2014). Patterns of infection in patients with myelodysplastic syndromes and acute myeloid leukemia receiving azacitidine as salvage therapy. Implications for primary antifungal prophylaxis. Clin Lymphoma Myeloma Leuk.

[31] Garcia CA, Wu S (2016). Attributable risk of infection to mTOR inhibitors Everolimus and Temsirolimus in the treatment of cancer. Cancer Investig.

[32] Garcia MDC, Redelman-Sidi G (2015). Opportunistic infections in patients receiving immunotherapy with CTLA-4, PD-1 and PD-L1 blockers for treatment of metastatic melanoma. Open Forum Infect Dis.

[33] Gardner A, Mattiuzzi G, Faderl S (2008). Randomized comparison of cooked and noncooked diets in patients undergoing remission induction therapy for acute myeloid leukemia. J Clin Oncol.

[34] Glasmacher A, Hahn C, Molitor E (1999). Itraconazole trough concentrations in antifungal prophylaxis with six different dosing regimens using hydroxypropyl-beta-cyclodextrin oral solution or coated-pellet capsules. Mycoses.

[35] Gomes MZ, Jiang Y, Mulanovich VE (2014). Effectiveness of primary anti-aspergillus prophylaxis during remission induction chemotherapy of acute myeloid leukemia. Antimicrob Agents Chemother.

[36] Gross BN, Ihorst G, Jung M (2013). Posaconazole therapeutic drug monitoring in the real-life setting: a single-center experience and review of the literature. Pharmacotherapy.

[37] Guinea J, Escribano P, Marcos-Zambrano LJ (2016). Therapeutic drug monitoring of voriconazole helps to decrease the percentage of patients with off-target trough serum levels. Med Mycol.

[38] Habermann TM, Weller EA, Morrison VA (2006). Rituximab-CHOP versus CHOP alone or with maintenance rituximab in older patients with diffuse large B-cell lymphoma. J Clin Oncol.

[39] Hachem R, Assaf A, Numan Y (2017). Comparing the safety and efficacy of voriconazole versus posaconazole in the prevention of invasive fungal infections in high-risk patients with hematological malignancies. Int J Antimicrob Agents.

[40] Hamada Y, Tokimatsu I, Mikamo H (2013). Practice guidelines for therapeutic drug monitoring of voriconazole: a consensus review of the Japanese Society of Chemotherapy and the Japanese Society of Therapeutic Drug Monitoring. J Infect Chemother.

[41] Heine A, Brossart P, Wolf D (2013). Ruxolitinib is a potent immunosuppressive compound: is it time for anti-infective prophylaxis?. Blood.

[42] Heine A, Held SE, Daecke SN (2013). The JAK-inhibitor ruxolitinib impairs dendritic cell function in vitro and in vivo. Blood.

[43] Heinz WJ, Buchheidt D, Christopeit M (2017). Diagnosis and empirical treatment of fever of unknown origin (FUO) in adult neutropenic patients: guidelines of the infectious diseases working party (AGIHO) of the German Society of Hematology and Medical Oncology (DGHO). Ann Hematol.

[44] Herbrecht R, Denning DW, Patterson TF (2002). Voriconazole versus amphotericin B for primary therapy of invasive aspergillosis. N Engl J Med.

[45] Howard SJ, Cerar D, Anderson MJ (2009). Frequency and evolution of azole resistance in aspergillus fumigatus associated with treatment failure. Emerg Infect Dis.

[46] Hu R, Jiang X, Wu Y (2013). Prospective trial finds nystatin mouthwash effective prophylaxis for pulmonary invasive fungal infections that originate in the throat of patients with hematologic malignancies. Neoplasma.

[47] Hussaini T, Ruping MJ, Farowski F (2011). Therapeutic drug monitoring of voriconazole and posaconazole. Pharmacotherapy.

[48] Imhof A, Schaer DJ, Schanz U (2006). Neurological adverse events to voriconazole: evidence for therapeutic drug monitoring. Swiss Med Wkly.

[49] Jang SH, Colangelo PM, Gobburu JV (2010). Exposure-response of posaconazole used for prophylaxis against invasive fungal infections: evaluating the need to adjust doses based on drug concentrations in plasma. Clin Pharmacol Ther.

[50] Johnson LB, Kauffman CA (2003). Voriconazole: a new triazole antifungal agent. Clin Infect Dis.

[51] Keating MJ, Flinn I, Jain V (2002). Therapeutic role of alemtuzumab (Campath-1H) in patients who have failed fludarabine: results of a large international study. Blood.

[52] Keighley CL, Manii P, Larsen SR (2017). Clinical effectiveness of itraconazole as antifungal prophylaxis in AML patients undergoing intensive chemotherapy in the modern era. Eur J Clin Microbiol Infect Dis.

[53] Koehler P, Hamprecht A, Bader O (2017). Epidemiology of invasive aspergillosis and azole resistance in patients with acute leukaemia: the SEPIA study. Int J Antimicrob Agents.

[54] Kontoyiannis DP, Marr KA, Park BJ (2010). Prospective surveillance for invasive fungal infections in hematopoietic stem cell transplant recipients, 2001-2006: overview of the transplant-associated infection surveillance network (TRANSNET) database. Clin Infect Dis.

[55] Kruger WH, Zollner B, Kaulfers PM (2003). Effective protection of allogeneic stem cell recipients against aspergillosis by HEPA air filtration during a period of construction--a prospective survey. J Hematother Stem Cell Res.

[56] Kuse E-R, Chetchotisakd P, Da Cunha CA (2007). Micafungin versus liposomal amphotericin B for candidaemia and invasive candidosis: a phase III randomised double-blind trial. Lancet.

[57] Kyi C, Hellmann MD, Wolchok JD (2014). Opportunistic infections in patients treated with immunotherapy for cancer. J Immunother Cancer.

[58] Lafon-DesmursB, MonselG, LeblondVet al.Sequential disseminated aspergillosis and pulmonary tuberculosis in a patient treated by idelalisib for chronic lymphocytic leukemia. Médecine et Maladies Infectieuses 10.1016/j.medmal.2016.10.001 27818019

[59] Larkin J, Hodi FS, Wolchok JD (2015). Combined Nivolumab and Ipilimumab or monotherapy in untreated melanoma. N Engl J Med.

[60] Laverdiere M, Bow EJ, Rotstein C (2014). Therapeutic drug monitoring for triazoles: a needs assessment review and recommendations from a Canadian perspective. Can J Infect Dis Med Microbiol.

[61] Lin P-C, Hsiao L-T, Poh S-B (2007). Higher fungal infection rate in elderly patients (more than 80years old) suffering from diffuse large B cell lymphoma and treated with rituximab plus CHOP. Ann Hematol.

[62] Marks DI, Liu Q, Slavin M (2017). Voriconazole for prophylaxis of invasive fungal infections after allogeneic hematopoietic stem cell transplantation. Expert Rev Anti-Infect Ther.

[63] Maschmeyer G (2006). The changing epidemiology of invasive fungal infections: new threats. Int J Antimicrob Agents.

[64] Maschmeyer G (2009). The changing face of febrile neutropenia-from monotherapy to moulds to mucositis. Prevention of mould infections. J Antimicrob Chemother.

[65] Maschmeyer G, Haas A, Cornely OA (2007). Invasive aspergillosis: epidemiology, diagnosis and management in immunocompromised patients. Drugs.

[66] Maschmeyer G, Neuburger S, Fritz L (2009). A prospective, randomised study on the use of well-fitting masks for prevention of invasive aspergillosis in high-risk patients. Ann Oncol.

[67] Menegueti MG, Ferreira LR, Silva MF (2013). Assessment of microbiological air quality in hemato-oncology units and its relationship with the occurrence of invasive fungal infections: an integrative review. Rev Soc Bras Med Trop.

[68] Merkel D, Filanovsky K, Gafter-Gvili A (2013). Predicting infections in high-risk patients with myelodysplastic syndrome/acute myeloid leukemia treated with azacitidine: a retrospective multicenter study. Am J Hematol.

[69] MoodyKM, BakerRA, SantizoROet al. (2017) A randomized trial of the effectiveness of the neutropenic diet versus food safety guidelines on infection rate in pediatric oncology patients. Pediatric Blood Cancer 10.1002/pbc.26711 28696047

[70] Mori Y, Ikeda K, Inomata T (2016). Ruxolitinib treatment for GvHD in patients with myelofibrosis. Bone Marrow Transplant.

[71] Morrison VA (2010). Infectious complications of chronic lymphocytic leukaemia: pathogenesis, spectrum of infection, preventive approaches. Best Pract Res Clin Haematol.

[72] Motzer RJ, Escudier B, Oudard S (2010). Phase 3 trial of everolimus for metastatic renal cell carcinoma : final results and analysis of prognostic factors. Cancer.

[73] Nachbaur D, Angelova O, Orth-Holler D (2015). Primary antifungal prophylaxis with micafungin in patients with haematological malignancies: real-life data from a retrospective single-centre observational study. Eur J Haematol.

[74] Nedel WL, Kontoyiannis DP, Pasqualotto AC (2009). Aspergillosis in patients treated with monoclonal antibodies. Revista Iberoamericana de Micologia.

[75] Neumann S, Krause SW, Maschmeyer G (2013). Primary prophylaxis of bacterial infections and pneumocystis jirovecii pneumonia in patients with hematological malignancies and solid tumors. Ann Hematol.

[76] Nihtinen A, Anttila VJ, Richardson M (2007). The utility of intensified environmental surveillance for pathogenic moulds in a stem cell transplantation ward during construction work to monitor the efficacy of HEPA filtration. Bone Marrow Transplant.

[77] Okamoto K, Proia LA, Demarais PL (2016). Disseminated Cryptococcal disease in a patient with chronic lymphocytic leukemia on Ibrutinib. Case Rep Infect Dis.

[78] Pagano L, Caira M, Candoni A (2006). The epidemiology of fungal infections in patients with hematologic malignancies: the SEIFEM-2004 study. Haematologica.

[79] Pascual A, Calandra T, Bolay S (2008). Voriconazole therapeutic drug monitoring in patients with invasive mycoses improves efficacy and safety outcomes. Clin Infect Dis.

[80] Pfaller MA, Castanheira M, Lockhart SR (2012). Frequency of decreased susceptibility and resistance to Echinocandins among fluconazole-resistant bloodstream isolates of Candida Glabrata. J Clin Microbiol.

[81] Pfaller MA, Diekema DJ (2007). Epidemiology of invasive candidiasis: a persistent public health problem. Clin Microbiol Rev.

[82] Pomares H, Arnan M, Sanchez-Ortega I (2016). Invasive fungal infections in AML/MDS patients treated with azacitidine: a risk worth considering antifungal prophylaxis?. Mycoses.

[83] Raad I, Hanna H, Osting C (2015). Masking of neutropenic patients on transport from hospital rooms is associated with a decrease in nosocomial aspergillosis during construction. Infect Control Hospital Epidemiol.

[84] Reinwald M, Boch T, Hofmann WK (2015). Risk of infectious complications in Hemato-oncological patients treated with kinase inhibitors. Biomark Insights.

[85] Ribera J-M, Ferrer A, Ribera J (2015). Profile of blinatumomab and its potential in the treatment of relapsed/refractory acute lymphoblastic leukemia. OncoTargets Therapy.

[86] Rieger CT, Cornely OA, Hoppe-Tichy T (2012). Treatment cost of invasive fungal disease (Ifd) in patients with acute myelogenous leukaemia (Aml) or myelodysplastic syndrome (Mds) in German hospitals. Mycoses.

[87] Rijnders BJ, Cornelissen JJ, Slobbe L (2008). Aerosolized liposomal amphotericin B for the prevention of invasive pulmonary aspergillosis during prolonged neutropenia: arandomized, placebo-controlled trial. Clin Infect Dis.

[88] Sarkaria JN, Galanis E, Wu W (2010). Combination of temsirolimus (CCI-779) with chemoradiation in newly diagnosed glioblastoma multiforme (GBM) (NCCTG trial N027D) is associated with increased infectious risks. Clin Cancer Res.

[89] Schelenz S, Hagen F, Rhodes JL (2016). First hospital outbreak of the globally emerging Candida Auris in a European hospital. Antimicrob Resist Infect Control.

[90] Schlesinger A, Paul M, Gafter-Gvili A (2009). Infection-control interventions for cancer patients after chemotherapy: a systematic review and meta-analysis. Lancet Infect Dis.

[91] Schönberg K, Rudolph J, Vonnahme M (2015). JAK inhibition impairs NK cell function in myeloproliferative neoplasms. Cancer Res.

[92] Schrenk KG, Schnetzke U, Stegemann K (2015). Efficacy of antifungal prophylaxis with oral suspension posaconazole during induction chemotherapy of acute myeloid leukemia. J Cancer Res Clin Oncol.

[93] Scodavolpe S, Quaranta S, Lacarelle B (2014). Triazole antifungal agents: practice guidelines of therapeutic drug monitoring and perspectives in treatment optimization. Ann Biol Clin.

[94] Stott KE, Hope WW (2017). Therapeutic drug monitoring for invasive mould infections and disease: pharmacokinetic and pharmacodynamic considerations. J Antimicrob Chemother.

[95] Tacke D, Buchheidt D, Karthaus M (2014). Primary prophylaxis of invasive fungal infections in patients with haematologic malignancies. 2014 update of the recommendations of the infectious diseases working Party of the German Society for Haematology and oncology. Ann Hematol.

[96] Tan K, Brayshaw N, Tomaszewski K (2006). Investigation of the potential relationships between plasma voriconazole concentrations and visual adverse events or liver function test abnormalities. J Clin Pharmacol.

[97] Teng JC, Slavin MA, Teh BW (2015). Epidemiology of invasive fungal disease in lymphoproliferative disorders. Haematologica.

[98] Topalian SL, Hodi FS, Brahmer JR (2012). Safety, activity, and immune correlates of anti-PD-1 antibody in cancer. N Engl J Med.

[99] Tortorano AM, Peman J, Bernhardt H (2004). Epidemiology of candidaemia in Europe: results of 28-month European Confederation of Medical Mycology (ECMM) hospital-based surveillance study. Eur J Clin Microbiol Infect Dis.

[100] Trifilio S, Ortiz R, Pennick G (2005). Voriconazole therapeutic drug monitoring in allogeneic hematopoietic stem cell transplant recipients. Bone Marrow Transplant.

[101] Trifilio S, Pennick G, Pi J (2007). Monitoring plasma voriconazole levels may be necessary to avoid subtherapeutic levels in hematopoietic stem cell transplant recipients. Cancer.

[102] Ueda K, Nannya Y, Kumano K (2009). Monitoring trough concentration of voriconazole is important to ensure successful antifungal therapy and to avoid hepatic damage in patients with hematological disorders. Int J Hematol.

[103] Ullmann AJ, Lipton JH, Vesole DH (2007). Posaconazole or fluconazole for prophylaxis in severe graft-versus-host disease. N Engl J Med.

[104] Ullmann AJ, Schmidt-Hieber M, Bertz H (2016). Infectious diseases in allogeneic haematopoietic stem cell transplantation: prevention and prophylaxis strategy guidelines 2016. Ann Hematol.

[105] Van Dalen EC, Mank A, Leclercq E (2016). Low bacterial diet versus control diet to prevent infection in cancer patients treated with chemotherapy causing episodes of neutropenia. Cochrane Database Syst Rev.

[106] Van Der Kolk LE, Baars JW, Prins MH (2002). Rituximab treatment results in impaired secondary humoral immune responsiveness. Blood.

[107] Van Tiel FH, Harbers MM, Terporten PHW (2007). Normal hospital and low-bacterial diet in patients with cytopenia after intensive chemotherapy for hematological malignancy: a study of safety†. Ann Oncol.

[108] Verweij PE, Ananda-Rajah M, Andes D (2015). International expert opinion on the management of infection caused by azole-resistant aspergillus fumigatus. Drug Resist Updates.

[109] Voog E, Morschhauser F, Solal-Celigny P (2003). Neutropenia in patients treated with rituximab. N Engl J Med.

[110] Weber JS, Kähler KC, Hauschild A (2012). Management of Immune-Related Adverse Events and Kinetics of response with Ipilimumab. J Clin Oncol.

[111] Wingard JR, Carter SL, Walsh TJ (2010). Randomized, double-blind trial of fluconazole versus voriconazole for prevention of invasive fungal infection after allogeneic hematopoietic cell transplantation. Blood.

[112] Wisplinghoff H, Ebbers J, Geurtz L (2014). Nosocomial bloodstream infections due to Candida spp. in the USA: species distribution, clinical features and antifungal susceptibilities. Int J Antimicrob Agents.

[113] Wysham NG, Sullivan DR, Allada G (2013). An opportunistic infection associated with ruxolitinib, a novel janus kinase 1,2 inhibitor. Chest.

[114] Xie S, Chen M, Yan B (2014). Identification of a role for the PI3K/AKT/mTOR signaling pathway in innate immune cells. PLoS One.

[115] Zonios DI, Gea-Banacloche J, Childs R (2008). Hallucinations during voriconazole therapy. Clin Infect Dis.

