# Supplementary material for: Primary prophylaxis of invasive fungal infections in patients with haematological malignancies: 2017 update of the recommendations of the Infectious Diseases Working Party (AGIHO) of the German Society for Haematology and Medical Oncology (DGHO)
Source: Ann Hematol. 2017 Dec 7;97(2):197–207. doi: 10.1007/s00277-017-3196-2 (PMC5754425; doi:10.1007/s00277-017-3196-2)
Supplement: Supplementary file 1 — (DOCX 232 kb) [file 277_2017_3196_MOESM1_ESM.docx]

**Appendix**

Table 5 – Fluconazole Prophylaxis

| **Author** | **Year** | **Design** | **Medication/daily dose** | **n=** | **Population** | **Invasive fungal infection** | | | **Mortality** | |
| --- | --- | --- | --- | --- | --- | --- | --- | --- | --- | --- |
|  |  |  | **per treatment arm** |  |  | **Proven** | **Probable** | **Possible** | **Total** | **Fungal** |
| Chandrasekar [1] | 1994 | rd, db, pl | FLU 400 mg placebo | 23 23 | 91% AL 9% other haematol | 8.6% 4.3% | 0% 0% | 0% 0% | 17.4% 13% | 8.6% 4.3% |
| Goodman [2] | 1992 | rd, db, pl | FLU 400 mg placebo | 179 177 | 48% alloSCT 52% autoSCT | 3% 16% | N/A | N/A | 30.7% 26% | 0.6% 5.6% |
| Rotstein, Laverdière [3, 4] | 1999 | rd, db, pl | FLU 400 mg placebo | 141 133 | 50% AML 10% ALL 31% other haematol 9% solid tumor 44% autoSCT | 2.8% 16.5% | 3.5% 7.5% | 36% 26% | 11% 11% | 0.7% 4.5% |
| Schaffner [5] | 1995 | rd, db, pl | FLU 400 mg placebo | 75 76 | 72% AML 28% NHL relapse 10% autoSCT | 8% 9.2% | 2.7% 1.3% | 6.6% 7.9% | 5.3% 6.6% | 2.6% 2.6% |
| Slavin, Marr [6, 7] | 1995 | rd, db, pl | FLU 400 mg placebo | 152 148 | 20% AML 10% ALL 55% NHL/HD 15% other 88% alloSCT 12% autoSCT | 7% 18% | 38% 55% | | 20% 35% | 13% 21% |
| Winston [8] | 1993 | rd, db, pl | FLU 400 mg placebo | 123 132 | 80% AML 20% ALL | 4% 8% | N/A | N/A | 0.8% 3% | 0% 0% |
| MacMillan [9] | 2002 | rd | FLU 400 mg FLU 200 mg | 124 129 | 36% AL 25% CML 17% NHL/HD 22% other 56% alloSCT 44% autoSCT | 7.3% 2.3% | N/A | N/A | N/A | N/A |
| Ullmann [10] | 2007 | rd, db | FLU 400 mg POS 600 mg | 299 301 | 100% alloSCT | 7% 2.3% | | 2% 3% | 28.1% 25.2% | 4% 1% |
| Cornely [11] | 2007 | rd | FLU 400 mg OR ITR 400 mg oral sol POS 600 mg | 298 304 | 86% AML 14% MDS | 8.4% 2.3% | |  | 22.5% 16.1% | 5.4% 1.7% |
| Winston [12] | 2003 | rd | FLU 400 mg i.v./p.o. ITR d1-2: 400 mg i.v., d3-: 200 mg i.v. or 400 mg sol | 67 71 | 100% alloSCT | 25.4% 8.5% | N/A | N/A | N/A | 17.9% 8.5% |
| Glasmacher [13] | 2006 | rd | FLU 400 mg sol ITR 5 mg/kg sol | 246 248 | 72.9% AML 12.1% ALL 15% other | 2% 1.6% | 11.4% 8.9% | | 11.4% 10.1% | 1.2% 0.8% |
| Marr [14] | 2004 | rd | FLU 400 mg i.v./p.o. ITR 200 mg i.v./ 7.5 mg/kg sol | 148 149 | 100% alloSCT | 16.2% 12.1% | | N/A | 39.2% 30.9% | 8% 7.4% |
| van Burik [15] | 2004 | rd, db, pl | FLU 400 mg i.v. MIC 50 mg | 457 425 | 14% AML 3% ALL 12% CML 24% NHL 20% MM 46% autoSCT 54% alloSCT | 26.5% 20% | | | 5.7% 4.2% | 0.4% 0.2% |
| Wingard [16] | 2010 | rd, db | FLU 400 mg VCZ 400 mg | 295  305 | 39% AML  20% ALL  17% CML  16% MDS  7% NHL  100% alloSCT | 3.1% 1.6% | 5.1% 2.6% | N/A | 20%  18.8% | N/A |
| Oren [17] | 2006 | rd | FLU 400 mg ITR 400 mg oral sol or ITR 200 mg IV | 99 96 | 31% AML 5% ALL 23% NHL 4% HD 55% autoSCT 19% alloSCT | 2% 1% | 6% 5.2% | 1% 3.1% | 11.1% 9.4% | 9.1% 5.2% |
| Mattiuzzi [18] | 2003 | rd | FLU 400 mg + ITR 400 mg cps L-AmB 3 mg/kg i.v. tiw | 67 70 | 100% AML | 4.5% 4.3% | N/A | 16% 8.6% | 11.9% 14.3% | 1.5% 1.4% |
| Yamac [19] | 1995 | rd | FLU 400 mg No prophylaxis | 41 29 | N/A | 9.8% 31% | | | N/A | N/A |
| Bodey [20] | 1994 | rd | FLU 400 mg AmB 0.5 mg/kg tiw i.v. | 41 36 | 94% AML 6% ALL | 4.8% 8.3% | 7.3% 19.4% | 4.8% 2.7% | 14.6% 25% | 0% 2.7% |
| Egger [21] | 1995 | rd | FLU 400 mg p.o./i.v. NYS 72 Mill IU + MCZ inh. (dose N/A) | 43 46 | 46% AL 16% NHL/HD 38% other | 2.3% 4.3% | | | N/A | 2.3% 0% |
| Kern [22] | 1998 | rd | FLU 400 mg + AmB 240 mg AmB 240 mg | 36 32 | 100% AML-Rez. | 5.6% 6.3% | 0 0 | 53% 34% | 22% 19% | 0% 0% |
| Wolff [23] | 2000 | rd | FLU 400 mg AmB 0.2 mg/kg i.v. | 196 159 | 103 alloSCT 252 autoSCT | 4.1% 7.5% | N/A | N/A | 12.2% 11.9% | 2.6% 1.3% |
| Hashino [24] | 2008 | hist contr | FLU 400 p.o. OR i.v. MIC 100 mg | 29 44 | 27% AML 19% ALL 54% other 100% alloSCT | 6.9% 0% | 3.4% 0% | 24.1% 11.4% | 10.3% 9.1% | 6.9% 0% |
| Annaloro [25] | 1995 | rd, hist contr | FLU 300 mg + NYS (dose N/A) FLU 50 mg + NYS (dose N/A) ITR 400 mg cps + NYS (dose N/A) | 28 30 31 | 35% alloSCT 65% autoSCT | 3.6% 3.3% 12.9% | 7.1% 6.6% 3.2% | | 7.1% 3.3% 6.4% | 0% 0% 0% |
| Ito [26] | 2007 | rd | FLU 200 mg ITR 200 mg cps | 110 108 | 100% AML/MDS | 0% 0% | 7.3% 0% | 2.7% 3.7% | N/A | N/A |
| Akayima [27] | 1993 | rd | FLU 200 mg AmB 2.4 g | 71 59 | 80% AML 18% ALL 2% NHL | 1.4% 3.4% | N/A | N/A | N/A | N/A |
| Young [28] | 1999 | rd, db, pl | FLU 200 mg NYS 6 Mill IU | 86 78 | 68% AML 25% ALL 7% other | 4.7% 7.7% | N/A | 16% 24% | 7% 14% | 2% 4% |
| Ellis [29] | 1994 | rd | FLU 200 mg CLO 20 mg + NYS 2 Mill IU | 42 48 | 47% AML 30% ALL 8% NHL/HD 16% other 26% alloSCT | 4.8% 21% | 2.3% 2.1% | N/A | N/A | 4.8% 18.8% |
| Meunier [30] | 1991 | hist contr | FLU 200 mg AmB 430 mg | 30 29 | 68% AL 22% NHL/HD 10% other | 13.3% 17.2% | N/A | N/A | 16.7% 27.6% | 3.3% 10.3% |
| Timmers [31] | 2000 | rd | FLU 200 mg ABCD 2 mg/kg i.v. | 12 12 | 25% AML 8% ALL 67% other haematol | 0 0 | N/A | N/A | 0% 17% | 0% 0% |
| Takatsuka [32] | 1999 | hist contr | FLU 200 mg + AmB sol 300 mg + AmB inh. (dose N/A) AmB 300 mg + AmB sol 300 mg + AmB inh. (dose N/A) | 54 70 | 46% AML 18% ALL 19% NHL 5% CML | 0 7% | N/A | N/A | N/A | N/A |
| Ninane [33] | 1994 | rd | FLU 3 mg/kg NYS 0.2 Mill IU/kg +/- AmB 100 mg/kg | 245 257 | 53% AL 12% NHL/HD 30% solid tumor 5% other | 0.8% 1.9% | N/A | N/A | 1.2% 2.3% | N/A |
| Groll [34] | 1997 | rd | FLU 3 mg/kg NYS 50.000 IU/kg | 25 25 | N/A | 4% 0% | 4% 8% | | N/A | N/A |
| Alangaden [35] | 1994 | hist contr | FLU 100/200 mg no prophylaxis | 112 79 | 50% AL 36% NHL/HD 15% other 67% alloSCT 33% autoSCT | 3.6% 10.1% | N/A | N/A | 8.9% 17.7% | 0.9% 3.8% |
| Menichetti [36] | 1994 | rd | FLU 150 mg AmB 2g sol | 420 400 | 81% AML 19% ALL | 2.6% 2.5% | N/A | 16% 21% | 10.5% 10% | 1.2% 0.8% |
| Huijgens [37] | 1999 | rd, db | FLU 100 mg ITR 200 mg cps | 101 101 | 39% AL 61% other haematol | 4% 4% | 7% 4% | 1% 2% | 7% 11% | 3% 6% |
| Morgenstern [38] | 1999 | rd | FLU 100 mg ITR 5 mg/kg sol | 293 288 | 53% AML 12% ALL 35% other haematol | 2% 0.3% | 2.4% 3.1% | | N/A | 2.4% 0 |
| Philpott-Howard, Brammer, Rozenberg-Arska [39-41] | 1993 | rd | FLU 50 mg AmB 2g sol or NYS 4 Mill IU | 256 255 | 76% AL 22% other haematol 2% solid tumour 21% alloSCT | 2.4% 3.5% | N/A | N/A | N/A | N/A |
| Finke [42] | 1990 | rd | FLU 50 mg AmB 800 mg | 19 21 | N/A | N/A | N/A | N/A | 15.8% 9.5% | N/A |
| Hiramatsu [43] | 2008 | rd | MIC 150 mg FLU 400 mg i.v. | 50 50 | 13% AML 6% ALL 9% MDS 22% MM 45% NHL/HD 5% other 48% autoSCT 52% alloSCT | 2.0% 2.0% | 0% 0% | 4% 12% | 8% 4% | 2% 2% |

Medication given p.o. and dosing interval qd unless otherwise stated, abs = abstract, AA = aplastic anaemia, ABCD = Amphotericin B colloidal dispersion, AmB = amphotericin B,L-AmB = liposomal amphotericin B, ABLC = amphotericin B lipid complex, AL = acute leukaemia, MPD = myeloproliferative disease, PNH = paroxysmal nocturnal haemoglobinuria, PSN = prolonged severe neutropenia CLPD = chronic lymphoproliferative disorders, CAS = caspofungin, CLO = clotrimazole, db = double blind, FLU = fluconazole, HEPA = high-efficiency particulate air filters, hist contr = historical control group, inh. = inhalation, ITR = itraconazole, LAF = laminar air-flow, N/A = data not available, KTC = ketoconazole; MCZ = miconazole, MIC = micafungin; sol = oral solution, sus = suspension, NYS = nystatin, pl = placebo controlled, rd = randomised, obs = observational, bid = twice daily, tid= thrice daily, biw = twice weekly, tiw = thrice weekly.

Table 6 – Itraconazole Prophylaxis

| **Author** | **Year** | **Design** | **Medication/daily dose** | **n=** | **Population** | **Invasive fungal infection** | | | **Mortality** | |
| --- | --- | --- | --- | --- | --- | --- | --- | --- | --- | --- |
|  |  |  | **per treatment arm** |  |  | **Proven** | **Probable** | **Possible** | **Total** | **Fungal** |
| Winston [12] | 2003 | rd | ITR d1-2: 400 mg i.v., d3-: 200 mg i.v. or 400 mg sol FLU 400 mg i.v./p.o. | 71 67 | 100% alloSCT | 8.5% 25.4% | N/A | N/A | N/A | 8.5% 17.9% |
| Mattiuzzi [44] | 2006 | rd | ITR 400 mg i.v. (d1-2), 200 mg i.v. (d3-) CAS 50 mg | 90 107 | 75% AML 25% MDS | 5.5% 6.5% | N/A | N/A | 7.8% 6.5% | 2.2% 3.7% |
| Oren [17] | 2006 | rd | ITR 400 mg oral sol or ITR 200 mg IV FLU 400 mg | 96 99 | 31% AML 5% ALL 23% NHL 4% HD 55% autoSCT 19% alloSCT | 1% 2% | 5.2% 6% | 3.1% 1% | 9.4% 11.1% | 5.2% 9.1 % |
| Marr [14] | 2004 | rd | ITR 200 mg i.v./ 7.5 mg/kg sol FLU 400 mg i.v./p.o. | 149 148 | 100% alloSCT | 12.1% 16.2% | | N/A | 30.9% 39.2% | 7.4% 8.0% |
| Cornely [11] | 2007 | rd | FLU 400 mg OR ITR 400 mg oral sol POS 600 mg | 298 304 | 86% AML 14% MDS | 8.4% 2.3% | |  | 22.5% 16.1% | 5.4% 1.7% |
| Lass-Flörl [45] | 2003 | rd | ITR 10 mg/kg sol AmB 3g | 56 59 | 49% AML 29% ALL 22% other | 1.8% 6.8% | 5.4% 5.1% | | 5.4% 5.1% | 0% 3.4% |
| Böhme [46] | 2000 | hist contr | ITR 400 mg sol AmB 0.5 mg/kg tiw i.v. | 72 61 | 87% AML 9% ALL 4% other | 16.7% 0% | 8.3% 9.3% | 4.1% 1.6% | N/A | N/A |
| Harousseau [47] | 2000 | rd, db, pl | ITR 5 mg/kg sol AmB 2 g cps | 281 276 | 57% AML 13% ALL 29% other | 2.8% 4.7% | 30% 29% | | 6% 8% | 0.4% 1.8% |
| Menichetti [48] | 1999 | rd, db, pl | ITR 5 mg sol + NYS 2 Mill. IU placebo + NYS 2 Mill. IU | 201 204 | 76% AL 24% other | 2.5% 4.4% | 21% 29% | | 7.5% 8.8% | 0.5% 2.5% |
| Glasmacher [13] | 2006 | rd | ITR 5 mg/kg sol FLU 400 mg sol | 248 246 | 72.9% AML 12.1% ALL 15% other | 1.6% 2% | 8.9% 11.4% | | 10.1% 11.4% | 0.8% 1.2% |
| Morgenstern [38] | 1999 | rd | ITR 5 mg/kg sol FLU 100 mg | 288 293 | 53% AML 12% ALL 35% other | 0.3% 2% | 3.1% 2.4% | | N/A | 0% 2.4%^¥^ |
| Boogaerts [49] | 2001 | rd | ITR 200 mg sol AmB 1.5 g + NYS 2 Mill IU | 144 133 | 66% AML 9% SCT | 4.9% 5.3% | 2.8% 3.8% | | 11.8% 12.8% | 4.2% 4.5% |
| Glasmacher [50] | 1998 | hist contr | ITR 400/600 mg cps + AmB Sol 2.4 g or NYS 24 Mill. IU AmB 2.4g sol or NYS 24 Mill. IU | 47 76 | ~85% AML ~15% ALL ~22% AL relapse | N/A | N/A | N/A | 17.9% 25% | 0.9% 8.8% |
| Mattiuzzi [18] | 2003 | rd | ITR 400 mg cps + FLU 400 mg L-AmB 3 mg/kg i.v. tiw | 67 70 | 100% AML | 4.5% 4.3% | N/A | 16% 8.6% | 11.9% 14.3% | 1.5% 1.4% |
| Kaptan [51] | 2003 | rd | ITR 400 mg cps no prophylaxis | 31 24 | 56% AML 44% ALL | 6.5% 8.3% | 6.5% 16.7% | N/A | 16.1% 8.3% | 0% 0% |
| Vreugdenhil [52] | 1993 | rd, db, pl | ITR 400 mg cps + AmB 4 g sol placebo + AmB 4g sol | 46 46 | 64% AML 24% ALL 12% other | 10.9% 19.6% | 4.3% 2.2% | 6.5% 10.9% | 21.7% 30.4% | 15.2% 15.2% |
| Annaloro [25] | 1995 | rd, hist contr | ITR 400 mg cps + NYS (dose N/A) FLU 300 mg + NYS (dose N/A) FLU 50 mg + NYS (dose N/A) | 31 28 30 | 35% alloSCT 65% autoSCT | 12.9% 3.6% 3.3% | 3.2% 7.1% 6.6% | | 6.4% 7.1% 3.3% | 0% 0% 0% |
| Böhme [53] | 1996 | hist contr | ITR 400 mg cps AmB 0.8 g p.o. | 241 223 | 65% AML 12% ALL 23% other | 4.6% 5.4% | 0.5% 0.9% | 1.5% 1.3% | N/A | N/A |
| Thunnissen [54] | 1991 | hist contr | ITR 400 mg cps NYS 10 Mill IU | 47 47 | 75% AML 25% ALL/NHL | 0 9% | N/A | N/A | 8.5% 26% | 0% 13% |
| Tricot [55] | 1987 | hist contr | ITR 400 mg cps KTC 400 mg | 45 52 | 68% AML 26% ALL 6% other | 24% 44.5% | 5.5% 8.5% | N/A | 24% 40.5% | 15.5% 36.5% |
| Lamy [56] | 1998 | non comparative | ITR 400 mg cps + LAF | 96 | 45% AML 10% ALL 45% other | 2% | 3% | N/A | N/A | 0% |
| Nucci [57] | 2000 | rd, db, pl | ITR 200 mg cps placebo | 104 106 | 60% AML 20% ALL 20% other | 4.8% 8.5% | N/A | N/A | 7.7% 6.6% | 1.9% 0.9% |
| Huijgens [37] | 1999 | rd, db | ITR 200 mg cps FLU 100 mg | 101 101 | 39% AL 61% other | 4% 4% | 4% 7% | 2% 1% | 11% 7% | 6% 3% |
| Ito [26] | 2007 | rd | ITR 200 mg cps FLU 200 mg | 108 110 | 100% AML/MDS | 0% 0% | 0% 7.3% | 3.7% 2.7% | N/A | N/A |
| Todeschini [58] | 1993 | hist contr | ITR 200 mg cps + AmB intranasal 10 mg no prophylaxis | 164 290 | 31% AML 13% ALL 41% NHL/HD 15% other 6% autoSCT | 2.4% 6.6% | 4.9% 7.6% | N/A | N/A | 1.2% 3.8% |
| Sánchez-Ortega [59] | 2011 | cohort | ITR 400 mg oral sol OR 200 mg i.v. POS 600 mg | 16 33 | 71.4% AML/MDS 6.1% ALL 8.2% CML 14.3% CLPD  100% alloSCT | 12,5% 0% | | N/A | 9% 37% | N/A |
| Huang [60] | 2012 | rd | MIC 50 mg i.v. ITR 5 mg/kg oral sol | 136 147 | 19% AML 16% ALL 14% CML  80% alloSCT 20% autoSCT | 0.7% 0% | 3.7% 1.4% | 5.9% 7.5% | 0% 0.7% | 0% 0% |
| Mattiuzzi [61] | 2009 | rd | VOR 600 mg i.v ITR 200 mg i.v. | 71 52 | 76% AML 24% MDS | 0% 4% | | N/A | 8.4% 11.5% | 0% 2% |
| Marks [62] | 2011 | rd | VOR 400 mg (tablets or oral sus) OR 8 mg/kg i.v. ITR 400 mg (oral sol or caps) OR 200 mg i.v. | 224 241 | 45% AML 18% ALL 14% MDS 19% NHL/HD 4% CML 1% other 100% alloSCT | 0.9% 0.4% | 0.4% 1.7% | N/A | 18.1% 19.1% | 0% 0.4% |
| Keighley [63] | 2017 | non comparative | ITR 200mg sol tid | 84 | 100% AML | 3.4% | | 6.3% | 11% | N/A |

For abbreviations refer to table 5.

Table 7 – Posaconazole and Voriconazole Prophylaxis

| **Author** | **Year** | **Design** | **Medication/daily dose** | **n=** | **Population** | **Invasive fungal infection** | | | | **Mortality** | | |
| --- | --- | --- | --- | --- | --- | --- | --- | --- | --- | --- | --- | --- |
|  |  |  | **per treatment arm** |  |  | **Proven** | **Probable** | **Possible** | | **Total** | | **Fungal** |
| Cornely [11] | 2007 | rd | POS 600 mg FLU 400 mg OR ITR 400 mg oral sol | 304 298 | 86% AML 14% MDS | 2.3% 8.4% | |  | | 16.1% 22.5% | | 1.7% 5.4% |
| Ullmann [10] | 2007 | rd, db | POS 600 mg FLU 400 mg | 301 299 | 100% alloSCT | 2.3% 7% | | 3% 2% | | 25.2% 28.1% | | 1% 4% |
| Vehreschild [64] | 2007 | rd, db, pl | VCZ 400 mg placebo | 10 15 | 100% AML | 0% 0% | 0% 26.7% | 0% 33% | | 0% 13.3% | | 0% 0% |
| Wingard [16] | 2010 | rd, db | FLU 400 mg VCZ 400 mg | 295  305 | 39% AML  20% ALL  17% CML  16% MDS  7% NHL  100% alloSCT | 3.1% 1.6% | 5.1% 2.6% | | N/A | | 20%  18.8% | N/A |
| Sánchez-Ortega [59] | 2011 | obs | ITR 400 mg oral sol OR 200 mg i.v. POS 600 mg | 16 33 | 71.4% AML/MDS 6.1% ALL 8.2% CML 14.3% CLPD 100% alloSCT | 12% 0% | | | N/A | | 9% 27% | N/A |
| Mattiuzzi [61] | 2009 | rd | VOR 600 mg i.v ITR 200 mg i.v. | 71 52 | 76% AML 24% MDS | 0% 4% | | | N/A | | 8.4% 11.5% | 0% 2% |
| Winston [65] | 2011 | non comparative | POS 600 mg | 106 | 57% AL 15% NHL/HD 12% MDS 7% CML 6% AA 3% other 100% alloSCT | 7.5% | N/A | | N/A | | 35% | 3.7% |
| Marks [62] | 2011 | rd | VOR 400 mg (tablets or oral sus) OR 8 mg/kg i.v. ITR 400 mg (oral sol or caps) OR 200 mg i.v. | 224 241 | 45% AML 18% ALL 14% MDS 19% NHL/HD 4% CML 1% other 100% alloSCT | 0.9% 0.4% | 0.4% 1.7% | | N/A | | 18.1% 19.1% | 0% 0.4% |
| Ananda-Rajah [66]^*^ | 2012 | re | FLU 400 mg ITR 5 mg/kg oral sol VOR 400 mg POS 600 mg | 36 49 58 67 | 91% AML 9% MDS | 17% 8.2% 1.7% 0% | | | 8% 7.8% 12.6% 3% | | N/A | 5.3% 3.4% 0%  N/A |
| Shen [67] | 2013 | rd | POS 600 mg FLU 400 mg | 117 117 | 89% AML 11% MDS | 3.4% 9.4% | | | 6% 12.8% | | 2.6% 6% | N/A |
| Torres [68] | 2010 | non comparative | VOR 400 mg oral OR i.v. | 127 | 100% prolonged severe neutropenia | 3.9% | | | N/A | | 7.8% | 0.7% |
| Chaftari [69] | 2012 | rd | ABLC 7.5 mg/kg qw POS 600 mg | 19 21 | 100% alloSCT | 5% 0% | 5% 0% | | N/A | | N/A | N/A |
| Duarte [70] | 2014 | uncontrolled | POS 200 mg tablet  POS 300 mg tablet | 19  32 | 74% AML  17% AML relapse  9% MDS | 5.3%  0% | 0%  0% | | 0%  0% | | 10.5%  6.3% | 0%  0% |
| Cornely [71] | 2016 | non comparative | POS 300 mg tablet | 210 | 46% AML  8% AML relapse  3% MDS  43% HSCT | <1% | | | 0% | | 9,5% | 0% |
| Cornely [72] | 2017 | rd | POS 300 mg bid iv d1, 300 mg/d iv d2-28, POS 400 mg bid oral sus or POS 200 mg tid oral sus | 237 | 64% AML  5% MDS  16% HSCT | 1% | | | 0% | | 8.0% | 0% |

For abbreviations refer to table 5; Some patients received more than one antifungal drug as prophylaxis; absolute numbers and percentages refer to patients receiving ≥7 days of azole prophylaxis

Table 8 – Ketoconazole, Miconazole and Clotrimazole Prophylaxis

| **Author** | **Year** | **Design** | **Medication/daily dose** | **n=** | **Population** | **Invasive fungal fnfection** | | | **Mortality** | |
| --- | --- | --- | --- | --- | --- | --- | --- | --- | --- | --- |
|  |  |  | **per treatment arm** |  |  | **Proven** | **Probable** | **Possible** | **Total** | **Fungal** |
| Brincker [73] | 1983 | rd, db, pl | KTC 400 mg placebo | 19 19 | 100% AL | 10.5% 10.5% | N/A | N/A | N/A | N/A |
| Hansen [74] | 1987 | rd, db, pl | KTC 400 mg placebo | 27 29 | 59% AL 9% NHL/HD 13% autoSCT 21% other | N/A | N/A | N/A | N/A | N/A |
| Donnelly [75] | 1984 | rd | KTC 400 mg AmB tab 400 mg and 70 mg loz KTC 400 mg + AmB tab 400 mg + 70 mg loz | 17 19 12 | 94% AL 6% alloBMT | 5.9% 5.3% 0% | N/A | 23.5% 10.5% 25.0% | 5.9% 5.3% 0% | 5.9% 5.3% 0% |
| Shepp [76] | 1985 | rd | KTC 400 mg NYS 3 Mill IU | 27 29 | 96% alloBMT 4% autoSCT | 0% 6.9% | N/A | N/A | 7.4% 17.2% | 0% 6.9% |
| Tricot [55] | 1987 | hist contr | KTC 400 mg ITR 400 mg cps | 52 45 | 68% AML 26% ALL 6% other | 44.5% 24% | 8.5% 5.5% | N/A | 40.5% 24% | 36.5% 15.5% |
| Vogler [77] | 1987 | rd | KTC 400 mg NYS 2 Mill IU | 22 24 | 89% AL | 22.7% 33.3% | 22.7% 16.7% | | N/A | N/A |
| Estey [78] | 1984 | rd | TMP 320 mg + SMX 1.6 g KTC 400 mg KTC 400 mg + TMP 320 mg + SMX 1.6 g no prophylaxis | 32 32 45 38 | N/A | 15.6% 0% 11.1% 2.6% | N/A | N/A | 21.9% 21.9% 13.3% 15.8% | 16% 0% 6.7% 0% |
| Hann [79] | 1982 | rd | KTC 200/400 mg AmB 40 mg loz + NYS 1 Mill IU | 37 35 | 83% AL 17% other | 0% 5.7% | N/A | 8.1% 5.7% | 10.8% 25.7% | 0% 5.7% |
| Jones [80] | 1984 | rd | KTC 200 mg  NYS 2 Mill IU | 18 18 | 72% AL 28% NHL/HD | 5.5% 0% | N/A | N/A | N/A | N/A |
| Palmblad [81] | 1992 | rd, db, pl | KTC 200 mg placebo | 50 57 | 100% AL | 6% 1.8% | N/A | N/A | N/A | 6% 0% |
| Brincker [82] | 1978 | rd, db, pl | MCZ 2 g p.o. placebo | 15 15 | 100% AL | 6.7% 13.3% | N/A | N/A | N/A | N/A |
| Egger [21] | 1995 | rd | NYS 72 Mill IU + MCZ inh. (dose N/A) FLU 400 mg p.o./i.v. | 46 43 | 46% AL 16% NHL/HD 38% other | 4.3% 2.3% | | | N/A | 0% 2.3% |
| Ellis [29] | 1994 | rd | CLO 20 mg + NYS 2 Mill IU FLU 200 mg | 48 42 | 77% AL 8% NHL/HD 16% other 26% alloSCT | 21% 4.8% | 2.1% 2.3% | N/A | N/A | 18.8% 4.8% |
| Cornely [83] | 2015 | non comparative | ISA i.v. 200 mg 2-1-1 d1, 200 mg 1-0-1 d2, 200 mg d3-28  ISA i.v. 400 mg 2-1-1 d1, 400 mg 1-0-1 d2, 400 mg d3-28 | 11  12 | 100% AML | 0%  0% | | 18.2%  0% | 0%  0% | 0%  0% |

For abbreviations refer to table 5.

Table 9 – Prophylaxis with Systemic Amphotericin B Deoxycholate and Lipid-Based Formulations

| **Author** | **Year** | **Design** | **Medication/daily dose** | **n=** | **Population** | **Invasive fungal infection** | | | **Mortality** | |
| --- | --- | --- | --- | --- | --- | --- | --- | --- | --- | --- |
|  |  |  | **per treatment arm** |  |  | **Proven** | **Probable** | **Possible** | **Total** | **Fungal** |
| Karthaus [84] | 1999 | hist contr | AmB 1 mg/kg/48h i.v. no prophylaxis | 104 104 | 100% AL | 0% 4.8% | 18.3% 34.6% | 12.5% 19.2% | N/A | N/A |
| Bodey [20] | 1994 | rd | AmB 0.5 mg/kg i.v. tiw FLU 400 mg | 36 41 | 94% AML 6% ALL | 8.3% 4.8% | 19.4% 7.3% | 2.7% 4.8% | 25% 14.6% | 2.7% 0% |
| Böhme [46] | 2000 | hist contr | AmB 0.5 mg/kg i.v. tiw ITR 400 mg sol | 61 72 | 87% AML 9% ALL 4% other | 0% 16.7% | 9.3% 8.3% | 1.6% 4.1% | N/A | N/A |
| Rousey [85] | 1991 | hist contr | AmB 20 mg i.v. + LAF LAF no prophylaxis | 110 48 28 | 100% alloSCT | 9% 23% 25% | N/A | N/A | 20% 35% 46% | 6% 13% 18% |
| Wolff [23] | 2000 | rd | AmB 0.2 mg/kg/d i.v. FLU 400 mg | 159 196 | 103 alloSCT 252 autoSCT | 7.5% 4.1% | N/A | N/A | 11.9% 12.2% | 1.3% 2.6% |
| Riley [86] | 1994 | rd, db, pl | AmB 0.1 mg/kg i.v. + LAF 82% placebo + LAF 44% | 17 18 | 86% haematol 14% solid tumor 69% alloSCT | 0 28% | N/A | 29% 44% | 0% 22% | 0% 11% |
| Perfect [87] | 1992 | rd, bl, pl | AmB 0.1 mg/kg i.v. + HEPA placebo + HEPA | 91 91 | 100% autoSCT 4% haematol 96% solid tumor | 1.1%  9.9% | N/A | N/A | 3.3% 12.1% | 0% 2.2% |
| Mattiuzzi [18] | 2003 | rd | L-AmB 3 mg/kg i.v. tiw FLU 400 mg + ITR cps 400 mg | 70 67 | 100% AML | 4.3% 4.5% | N/A | 8.6% 16% | 14.3% 11.9% | 1.4% 1.5% |
| Kelsey [88] | 1999 | rd, db, pl | L-AmB 2 mg/kg i.v. tiw placebo | 74 87 | 27% AML 12% ALL 34% CML 22% NHL/HD 5% other 53% alloSCT 31% autoSCT | 0 2.3% | 28.3% 35.6% | | 15% 14% | 2.7% 2.3% |
| Tollemar [89, 90] | 1993 | rd, db, pl | L-AmB 1.0 mg/kg i.v. placebo | 36 40 | 25% AML 22% ALL 42% other 5% solid tumor 83% alloSCT | 3% 8% | 14% 18% | | 44% 36% | 3% 8% |
| Penack [91] | 2006 | rd | L-AmB 50 mg q48h no prophylaxis | 75 57 | 65% AML 14% ALL 20% NHL | 6.7% 35.1% | | N/A | 5.3% 14% | 2.7% 12.3% |
| Cordonnier [92] | 2008 | non comparative | L-AmB 10 mg/kg qwk | 29 | 69% AML 17% ALL 28% alloSCT | 3.4% | 10.3% | N/A | 13.8% | 3.4% |
| El-Cheikh [93] | 2007 | non comparative | L-AmB 7.5 mg/kg qwk | 21 | 100% alloSCT | N/A | 4.8% | | 38.1% | 0% |
| Timmers [31] | 2000 | rd | ABCD 2 mg/kg i.v. FLU 200 mg | 12 12 | 25% AML 8% ALL 67% other haematol | 0% 0% | N/A | N/A | 17% 0% | 0% 0% |
| Chaftari [69] | 2012 | rd | ABLC 7.5 mg/kg qw POS 600 mg | 19 21 | 100% alloSCT | 5% 0% | 5% 0% | N/A | N/A | N/A |
| Annino [94] | 2013 | non comparative | L-AmB 15 mg/kg single infusion, second dose after 15 days of persistent neutropenia | 48 | 100% AML | 8.3% | N/A | 4.2% | 27.1% | 8.3% |
| Cornely [95] | 2017 | rd | L-AmB 5 mg/kg i.v.  placebo | 237  118 | 100% ALL | 7.9%  11.7% | | 4.8%  5.4% | 7.2%  6.8% | 0,8%  0% |

For abbreviations refer to table 5.

Table 10 – Prophylactic Amphotericin B Inhalation

| **Author** | **Year** | **Design** | **Medication/daily dose** | **n=** | **Population** | **Invasive fungal infection** | | | **Mortality** | |
| --- | --- | --- | --- | --- | --- | --- | --- | --- | --- | --- |
|  |  |  | **per treatment arm** |  |  | **Proven** | **Probable** | **Possible** | **Total** | **Fungal** |
| Rijnders [96] | 2008 | rd, db, pl | L-AmB 12.5 mg inh. + FLU (dose N/A) placebo inh. + FLU (dose N/A) | 139 132 | 49% AML/MDS 21% autoSCT 11% alloSCT | 4.3% 13.6% | | N/A | 2.9%* 3.0%* | 3.6%** 4.5%** |
| Alexander [97] | 2006 | non comparative | ABLC 50 mg inh. d1-4, then qwk + FLU p.o./i.v. d1-100 | 40 | 100% alloSCT | 7.5% | 0% | 0% | 30% | 2.5% |
| Erjavec [98] | 1997 | non comparative | AmB 30 mg inh. | 42 | 52% AML 41% ALL 7% other haematol | 14% | 14% | N/A | N/A | 7.1% |
| Schwartz [99] | 1999 | rd | AmB 20 mg inh. no prophylaxis | 227 155 | 74% AML 9% ALL/NHL relapse 17% autoSCT | 1.8% 0.6% | 2.2% 5.8% | 0.4% 0.6% | 13% 10% | 8% 7% |
| Hertenstein [100] | 1994 | non comparative | AmB 20 mg inh. | 303 | 55% AML 26% CML 19% other 89% alloSCT 9% autoSCT | 3.6% | N/A | N/A | 20.5% | 2.6% |
| Takatsuka [32] | 1999 | hist contr | AmB 300 mg + AmB sol 300 mg + inh. (dose N/A) FLU 200 mg + AmB sol 300 mg + AmB inh. (dose N/A) | 70 54 | 46% AML 18% ALL 19% NHL 5% CML | 7% 0% | N/A | N/A | N/A | N/A |
| Hullard-Pulstinger [101] | 2011 | hist contr | L-AmB 12.5 mg inh. + FLU 400 mg placebo inh. + FLU 400 mg | 98 118 | 67% AML, MDS, AL  13% ALL 11% NHL/HD/MM 7% MPD 1% AA, PNH 34% alloSCT | 0% 2.5% | 2% 1% | N/A | N/A | N/A |
| Chong [102] | 2015 | hist contr | L-AmB 12.5 mg inh. + FLU 400 mg po  FLU 400mg po | 127  108 | 86% AML  13% MDS  < 1% CML | 9.5%  23.4% | | 4.8%  8.4% | 18.1%  17.6% | 2.4%  1.9% |

For abbreviations refer to table 5, * at 28 days, ** at 24 weeks.

Table 11 – Caspofungin and Micafungin Prophylaxis

| **Author** | **Year** | **Design** | **Medication/daily dose** | **n=** | **Population** | **Invasive fungal infection** | | | **Mortality** | |
| --- | --- | --- | --- | --- | --- | --- | --- | --- | --- | --- |
|  |  |  | **per treatment arm** |  |  | **Proven** | **Probable** | **Possible** | **Total** | **Fungal** |
| van Burik [15] | 2004 | rd, db, pl | MIC 50 mg FLU 400 mg i.v. | 425 457 | 14% AML 3% ALL 12% CML 24% NHL 20% MM 46% autoSCT 54% alloSCT | 20% 26.5% | | | 4.2% 5.7% | 0.2% 0.4% |
| Hashino [24] | 2008 | hist contr | MIC 100 mg  FLU 400 p.o. OR i.v. | 44 29 | 27% AML 19% ALL 54% other 100% alloSCT | 0% 6.9% | 0% 3.4% | 11.4% 24.1% | 9.1% 10.3% | 0% 6.9% |
| Mattiuzzi [44] | 2006 | rd | CAS 50 mg ITR 400 mg i.v. (d1-2), 200 mg i.v. (d3-) | 107 90 | 75% AML 25% MDS | 6.5% 5.5% | N/A | N/A | 6.5% 7.8% | 3.7% 2.2% |
| Hiramatsu [43] | 2008 | rd | MIC 150 mg i.v.  FLU 400 mg i.v. | 50  50 | 13% AML  6% ALL  9% MDS  22% MM  45% NHL/HD  5% other  48% autoSCT  52% alloSCT | 2.0%  2.0% | 0  0 | 4%  12% | 8%  4% | 2%  2% |
| Cattaneo [103] | 2011 | rd | CAS 50 mg ITR, FLU, POS, or no prophylaxis | 93 82 | 79% AML 21% ALL | 7.5% 3.7% | | 8.6% 17.1% | 9.7% 7.3% | 1% 0% |
| Huang [60] | 2012 | rd | MIC 50 mg i.v. ITR 5 mg/kg oral sol | 136 147 | 19% AML 16% ALL 14% CML  80% alloSCT 20% autoSCT | 0.7% 0% | 3.7% 1.4% | 5.9% 7.5% | 0% 0.7% | 0% 0% |
| Nachbaur [104] | 2015 | hist contr | MIC 50 mg i.v.  POS 200 mg tid | 100  202 | 37% AML  7% ALL  38% alloSCT | 6.0%  5.4% | 3.0%  7.9% | N/A  N/A | 33%  26% | N/A  N/A |

For abbreviations refer to table 5.

**References**

1. Chandrasekar, P.H. and C.M. Gatny, *Effect of fluconazole prophylaxis on fever and use of amphotericin in neutropenic cancer patients. Bone Marrow Transplantation Team.* Chemotherapy, 1994. **40**(2): p. 136-43.

2. Goodman, J.L., et al., *A controlled trial of fluconazole to prevent fungal infections in patients undergoing bone marrow transplantation.* N Engl J Med, 1992. **326**(13): p. 845-51.

3. Rotstein, C., et al., *Randomized placebo-controlled trial of fluconazole prophylaxis for neutropenic cancer patients: benefit based on purpose and intensity of cytotoxic therapy. The Canadian Fluconazole Prophylaxis Study Group.* Clin Infect Dis, 1999. **28**(2): p. 331-40.

4. Laverdiere, M., et al., *Impact of fluconazole prophylaxis on fungal colonization and infection rates in neutropenic patients. The Canadian Fluconazole Study.* J Antimicrob Chemother, 2000. **46**(6): p. 1001-8.

5. Schaffner, A. and M. Schaffner, *Effect of prophylactic fluconazole on the frequency of fungal infections, amphotericin B use, and health care costs in patients undergoing intensive chemotherapy for hematologic neoplasias.* J Infect Dis, 1995. **172**(4): p. 1035-41.

6. Slavin, M.A., et al., *Efficacy and safety of fluconazole prophylaxis for fungal infections after marrow transplantation--a prospective, randomized, double-blind study.* J Infect Dis, 1995. **171**(6): p. 1545-52.

7. Marr, K.A., et al., *Prolonged fluconazole prophylaxis is associated with persistent protection against candidiasis-related death in allogeneic marrow transplant recipients: long-term follow-up of a randomized, placebo-controlled trial.* Blood., 2000. **96**(6): p. 2055-61.

8. Winston, D.J., et al., *Fluconazole prophylaxis of fungal infections in patients with acute leukemia. Results of a randomized placebo-controlled, double-blind, multicenter trial.* Ann Intern Med, 1993. **118**(7): p. 495-503.

9. MacMillan, M.L., et al., *Fluconazole to prevent yeast infections in bone marrow transplantation patients: a randomized trial of high versus reduced dose, and determination of the value of maintenance therapy.* Am J Med, 2002. **112**(5): p. 369-79.

10. Ullmann, A.J., et al., *Posaconazole or fluconazole for prophylaxis in severe graft-versus-host disease.* N Engl J Med, 2007. **356**(4): p. 335-47.

11. Cornely, O.A., et al., *Posaconazole vs. fluconazole or itraconazole prophylaxis in patients with neutropenia.* N Engl J Med, 2007. **356**(4): p. 348-59.

12. Winston, D.J., et al., *Intravenous and oral itraconazole versus intravenous and oral fluconazole for long-term antifungal prophylaxis in allogeneic hematopoietic stem-cell transplant recipients. A multicenter, randomized trial.* Ann Intern Med, 2003. **138**(9): p. 705-13.

13. Glasmacher, A., et al., *An open-label randomized trial comparing itraconazole oral solution with fluconazole oral solution for primary prophylaxis of fungal infections in patients with haematological malignancy and profound neutropenia.* J Antimicrob Chemother, 2006. **57**(2): p. 317-25.

14. Marr, K.A., et al., *Itraconazole versus fluconazole for prevention of fungal infections in patients receiving allogeneic stem cell transplants.* Blood, 2004. **103**(4): p. 1527-33.

15. van Burik, J.A., et al., *Micafungin versus fluconazole for prophylaxis against invasive fungal infections during neutropenia in patients undergoing hematopoietic stem cell transplantation.* Clin Infect Dis, 2004. **39**(10): p. 1407-16.

16. Wingard, J.R., et al., *Randomized, double-blind trial of fluconazole versus voriconazole for prevention of invasive fungal infection after allogeneic hematopoietic cell transplantation.* Blood, 2010. **116**(24): p. 5111-8.

17. Oren, I., et al., *A prospective randomized trial of itraconazole vs fluconazole for the prevention of fungal infections in patients with acute leukemia and hematopoietic stem cell transplant recipients.* Bone Marrow Transplant, 2006. **38**(2): p. 127-34.

18. Mattiuzzi, G.N., et al., *Liposomal amphotericin B versus the combination of fluconazole and itraconazole as prophylaxis for invasive fungal infections during induction chemotherapy for patients with acute myelogenous leukemia and myelodysplastic syndrome.* Cancer, 2003. **97**(2): p. 450-6.

19. Yamac, K., E. Senol, and R. Haznedar, *Prophylactic use of fluconazole in neutropenic cancer patients.* Postgrad Med J, 1995. **71**(835): p. 284-6.

20. Bodey, G.P., et al., *Antifungal prophylaxis during remission induction therapy for acute leukemia fluconazole versus intravenous amphotericin B.* Cancer, 1994. **73**(8): p. 2099-106.

21. Egger, T., et al., *Comparison of fluconazole with oral polyenes in the prevention of fungal infections in neutropenic patients. A prospective, randomized, single-center study.* Support Care Cancer, 1995. **3**(2): p. 139-46.

22. Kern, W., et al., *Failure of fluconazole prophylaxis to reduce mortality or the requirement of systemic amphotericin B therapy during treatment for refractory acute myeloid leukemia: results of a prospective randomized phase III study. German AML Cooperative Group.* Cancer, 1998. **83**(2): p. 291-301.

23. Wolff, S.N., et al., *Fluconazole vs low-dose amphotericin B for the prevention of fungal infections in patients undergoing bone marrow transplantation: a study of the North American Marrow Transplant Group.* Bone Marrow Transplant, 2000. **25**(8): p. 853-9.

24. Hashino, S., et al., *Administration of micafungin as prophylactic antifungal therapy in patients undergoing allogeneic stem cell transplantation.* Int J Hematol, 2008. **87**(1): p. 91-7.

25. Annaloro, C., et al., *Efficacy of different prophylactic antifungal regimens in bone marrow transplantation.* Haematologica, 1995. **80**(6): p. 512-7.

26. Ito, Y., et al., *The prophylactic effect of itraconazole capsules and fluconazole capsules for systemic fungal infections in patients with acute myeloid leukemia and myelodysplastic syndromes: a Japanese multicenter randomized, controlled study.* Int J Hematol, 2007. **85**(2): p. 121-7.

27. Akiyama, H., et al., *Fluconazole versus oral amphotericin B in preventing fungal infection in chemotherapy-induced neutropenic patients with haematological malignancies.* Mycoses, 1993. **36**(11-12): p. 373-8.

28. Young, G.A., et al., *A double-blind comparison of fluconazole and nystatin in the prevention of candidiasis in patients with leukaemia. Antifungal Prophylaxis Study Group.* Eur J Cancer, 1999. **35**(8): p. 1208-13.

29. Ellis, M.E., et al., *Controlled study of fluconazole in the prevention of fungal infections in neutropenic patients with haematological malignancies and bone marrow transplant recipients.* Eur J Clin Microbiol Infect Dis, 1994. **13**(1): p. 3-11.

30. Meunier, F., et al., *Chemoprophylaxis of fungal infections in granulocytopenic patients using fluconazole vs oral amphotericin B.* Drug Invest, 1991. **3**(4): p. 258-265.

31. Timmers, G.J., et al., *Amphotericin B colloidal dispersion (Amphocil) vs fluconazole for the prevention of fungal infections in neutropenic patients: data of a prematurely stopped clinical trial.* Bone Marrow Transplant, 2000. **25**(8): p. 879-84.

32. Takatsuka, H., et al., *Fluconazole versus amphotericin B for the prevention of fungal infection in neutropenic patients with hematologic malignancy.* Drugs Exp Clin Res, 1999. **25**(4): p. 193-200.

33. Ninane, J., *A multicentre study of fluconazole versus oral polyenes in the prevention of fungal infection in children with hematological or oncological malignancies. Multicentre Study Group.* Eur J Clin Microbiol Infect Dis, 1994. **13**(4): p. 330-7.

34. Groll, A.H., et al., *Fluconazole versus nystatin in the prevention of candida infections in children and adolescents undergoing remission induction or consolidation chemotherapy for cancer.* J Antimicrob Chemother, 1997. **40**(6): p. 855-62.

35. Alangaden, G., et al., *Antifungal prophylaxis with low-dose fluconazole during bone marrow transplantation. The Bone Marrow Transplantation Team.* Bone Marrow Transplant, 1994. **14**(6): p. 919-24.

36. Menichetti, F., et al., *Preventing fungal infection in neutropenic patients with acute leukemia: fluconazole compared with oral amphotericin B. The GIMEMA Infection Program.* Ann Intern Med, 1994. **120**(11): p. 913-8.

37. Huijgens, P.C., et al., *Fluconazole versus itraconazole for the prevention of fungal infections in haemato-oncology.* J Clin Pathol, 1999. **52**(5): p. 376-80.

38. Morgenstern, G.R., et al., *A randomized controlled trial of itraconazole versus fluconazole for the prevention of fungal infections in patients with haematological malignancies. U.K. Multicentre Antifungal Prophylaxis Study Group.* Br J Haematol, 1999. **105**(4): p. 901-11.

39. Philpott-Howard, J.N., et al., *Randomized comparison of oral fluconazole versus oral polyenes for the prevention of fungal infection in patients at risk of neutropenia. Multicentre Study Group.* J Antimicrob Chemother, 1993. **31**(6): p. 973-84.

40. Brammer, K.W., *Management of fungal infection in neutropenic patients with fluconazole.* Haematology and Blood Transfusion, 1990. **33**: p. 546-550.

41. Rozenberg-Arska, M., et al., *A randomized study to compare oral fluconazole to amphotericin B in the prevention of fungal infections in patients with acute leukaemia.* J Antimicrob Chemother, 1991. **27**(3): p. 369-76.

42. Finke, R., *[Comparison of oral fluconazole and amphotericin B prophylaxis against fungal infections in the neutropenic phase of patients treated with antileukemic agents].* Mycoses, 1990. **33 Suppl 1**: p. 42-54.

43. Hiramatsu, Y., et al., *Use of micafungin versus fluconazole for antifungal prophylaxis in neutropenic patients receiving hematopoietic stem cell transplantation.* Int J Hematol, 2008. **88**(5): p. 588-95.

44. Mattiuzzi, G.N., et al., *Open-label, randomized comparison of itraconazole versus caspofungin for prophylaxis in patients with hematologic malignancies.* Antimicrob Agents Chemother, 2006. **50**(1): p. 143-7.

45. Lass-Flörl, C., et al., *Fungal colonization in neutropenic patients: a randomized study comparing itraconazole solution and amphotericin B solution.* Ann Hematol, 2003. **DOI 10.1007/s00277-003-0666-5, Published online: 21 June 2003**.

46. Böhme, A. and D. Hoelzer, *Primary antifungal prophylaxis with low-dose intravenous amphotericin B in hematological malignancies. Results of a pilot study.* Onkologie, 2000. **23**: p. 145-150.

47. Harousseau, J.L., et al., *Itraconazole oral solution for primary prophylaxis of fungal infections in patients with hematological malignancy and profound neutropenia: a randomized, double-blind, double-placebo, multicenter trial comparing itraconazole and amphotericin B.* Antimicrob Agents Chemother, 2000. **44**(7): p. 1887-93.

48. Menichetti, F., et al., *Itraconazole oral solution as prophylaxis for fungal infections in neutropenic patients with hematologic malignancies: a randomized, placebo-controlled, double-blind, multicenter trial. GIMEMA Infection Program. Gruppo Italiano Malattie Ematologiche dell' Adulto.* Clin Infect Dis, 1999. **28**(2): p. 250-5.

49. Boogaerts, M., et al., *Itraconazole versus amphotericin B plus nystatin in the prophylaxis of fungal infections in neutropenic cancer patients.* J Antimicrob Chemother, 2001. **48**(1): p. 97-103.

50. Glasmacher, A., et al., *Antifungal prophylaxis with itraconazole in neutropenic patients with acute leukaemia.* Leukemia, 1998. **12**(9): p. 1338-43.

51. Kaptan, K., et al., *Itraconazole is not effective for the prophylaxis of fungal infections in patients with neutropenia.* J Infect Chemother, 2003. **9**(1): p. 40-5.

52. Vreugdenhil, G., et al., *Efficacy of itraconazole in the prevention of fungal infections among neutropenic patients with hematologic malignancies and intensive chemotherapy. A double blind, placebo controlled study.* Leuk Lymphoma, 1993. **11**(5-6): p. 353-8.

53. Böhme, A., et al., *Itraconazole for prophylaxis of systemic mycoses in neutropenic patients with haematological malignancies.* J Antimicrob Chemother, 1996. **38**(6): p. 953-61.

54. Thunnissen, P.L., W. Sizoo, and W.D. Hendriks, *Safety and efficacy of itraconazole in prevention of fungal infections in neutropenic patients.* Neth J Med, 1991. **39**(1-2): p. 84-91.

55. Tricot, G., et al., *Ketoconazole vs. itraconazole for antifungal prophylaxis in patients with severe granulocytopenia: preliminary results of two nonrandomized studies.* Rev Infect Dis, 1987. **9 Suppl 1**: p. S94-9.

56. Lamy, T., et al., *Prophylactic use of itraconazole for the prevention of invasive pulmonary aspergillosis in high risk neutropenic patients.* Leuk Lymphoma, 1998. **30**(1-2): p. 163-74.

57. Nucci, M., et al., *A double-blind, randomized, placebo-controlled trial of itraconazole capsules as antifungal prophylaxis for neutropenic patients.* Clin Infect Dis, 2000. **30**(2): p. 300-5.

58. Todeschini, G., et al., *Oral itraconazole plus nasal amphotericin B for prophylaxis of invasive aspergillosis in patients with hematological malignancies.* Eur J Clin Microbiol Infect Dis, 1993. **12**(8): p. 614-8.

59. Sanchez-Ortega, I., et al., *Clinical efficacy and safety of primary antifungal prophylaxis with posaconazole vs itraconazole in allogeneic blood and marrow transplantation.* Bone Marrow Transplant, 2011. **46**(5): p. 733-9.

60. Huang, X., et al., *Multicenter, randomized, open-label study comparing the efficacy and safety of micafungin versus itraconazole for prophylaxis of invasive fungal infections in patients undergoing hematopoietic stem cell transplant.* Biol Blood Marrow Transplant, 2012. **18**(10): p. 1509-16.

61. Mattiuzzi, G.N., et al., *Efficacy and safety of intravenous voriconazole and intravenous itraconazole for antifungal prophylaxis in patients with acute myelogenous leukemia or high-risk myelodysplastic syndrome.* Support Care Cancer, 2011. **19**(1): p. 19-26.

62. Marks, D.I., et al., *Voriconazole versus itraconazole for antifungal prophylaxis following allogeneic haematopoietic stem-cell transplantation.* Br J Haematol, 2011. **155**(3): p. 318-27.

63. Keighley, C.L., et al., *Clinical effectiveness of itraconazole as antifungal prophylaxis in AML patients undergoing intensive chemotherapy in the modern era.* Eur J Clin Microbiol Infect Dis, 2017. **36**(2): p. 213-217.

64. Vehreschild, J.J., et al., *A double-blind trial on prophylactic voriconazole (VRC) or placebo during induction chemotherapy for acute myelogenous leukaemia (AML).* J Infect, 2007. **55**(5): p. 445-9.

65. Winston, D.J., et al., *Efficacy, safety, and breakthrough infections associated with standard long-term posaconazole antifungal prophylaxis in allogeneic stem cell transplantation recipients.* Biol Blood Marrow Transplant, 2011. **17**(4): p. 507-15.

66. Ananda-Rajah, M.R., et al., *Comparative clinical effectiveness of prophylactic voriconazole/posaconazole to fluconazole/itraconazole in patients with acute myeloid leukemia/myelodysplastic syndrome undergoing cytotoxic chemotherapy over a 12-year period.* Haematologica, 2012. **97**(3): p. 459-63.

67. Shen, Y., et al., *Posaconazole vs. fluconazole as invasive fungal infection prophylaxis in China: a multicenter, randomized, open-label study.* Int J Clin Pharmacol Ther, 2013. **51**(9): p. 738-45.

68. Torres, A., et al., *Voriconazole as primary antifungal prophylaxis in patients with neutropenia after hematopoietic stem cell transplantation or chemotherapy for acute myeloid leukemia.* Eur J Haematol, 2010. **84**(3): p. 271-3.

69. Chaftari, A.M., et al., *Comparison of posaconazole versus weekly amphotericin B lipid complex for the prevention of invasive fungal infections in hematopoietic stem-cell transplantation.* Transplantation, 2012. **94**(3): p. 302-8.

70. Duarte, R.F., et al., *Phase 1b study of new posaconazole tablet for prevention of invasive fungal infections in high-risk patients with neutropenia.* Antimicrob Agents Chemother, 2014. **58**(10): p. 5758-65.

71. Cornely, O.A., et al., *Phase 3 pharmacokinetics and safety study of a posaconazole tablet formulation in patients at risk for invasive fungal disease.* J Antimicrob Chemother, 2016. **71**(3): p. 718-26.

72. O.A. Cornely, e.a., *Pharmacokinetics and safety results from the Phase 3 randomized, open-label, study of intravenous posaconazole in patients at risk of invasive fungal disease.* Journal of Antimicrobial Chemotherapy, 2017.

73. Brincker, H., *Prevention of mycosis in granulocytopenic patients with prophylactic ketoconazole treatment.* Mykosen, 1983. **26**(5): p. 242-7.

74. Hansen, R.M., et al., *Ketoconazole in the prevention of candidiasis in patients with cancer. A prospective, randomized, controlled, double-blind study.* Arch Intern Med, 1987. **147**(4): p. 710-2.

75. Donnelly, J.P., et al., *Oral ketoconazole and amphotericin B for the prevention of yeast colonization in patients with acute leukaemia.* J Hosp Infect, 1984. **5**(1): p. 83-91.

76. Shepp, D.H., et al., *Comparative trial of ketoconazole and nystatin for prevention of fungal infection in neutropenic patients treated in a protective environment.* J Infect Dis, 1985. **152**(6): p. 1257-63.

77. Vogler, W.R., L.G. Malcom, and E.F. Winton, *A randomized trial comparing ketoconazole and nystatin prophylactic therapy in neutropenic patients.* Cancer Invest, 1987. **5**(4): p. 267-73.

78. Estey, E., et al., *Infection prophylaxis in acute leukemia. Comparative effectiveness of sulfamethoxazole and trimethoprim, ketoconazole, and a combination of the two.* Arch Intern Med, 1984. **144**(8): p. 1562-8.

79. Hann, I.M., et al., *Ketoconazole versus nystatin plus amphotericin B for fungal prophylaxis in severely immunocompromised patients.* Lancet, 1982. **1**(8276): p. 826-9.

80. Jones, P.G., et al., *Efficacy of ketoconazole v nystatin in prevention of fungal infections in neutropenic patients.* Arch Intern Med, 1984. **144**(3): p. 549-51.

81. Palmblad, J., et al., *Oral ketoconazole prophylaxis for Candida infections during induction therapy for acute leukaemia in adults: more bacteraemias.* J Intern Med, 1992. **231**(4): p. 363-70.

82. Brincker, H., *Prophylactic treatment with miconazole in patients highly predisposed to fungal infection. A placebo-controlled double-blind study.* Acta Med Scand, 1978. **204**(1-2): p. 123-8.

83. Cornely, O.A., et al., *Safety and pharmacokinetics of isavuconazole as antifungal prophylaxis in acute myeloid leukemia patients with neutropenia: results of a phase 2, dose escalation study.* Antimicrob Agents Chemother, 2015. **59**(4): p. 2078-85.

84. Karthaus, M., et al., *Intensive intravenous amphotericin B for prophylaxis of systemic fungal infections. Results of a prospective controlled pilot study in acute leukemia patients.* Chemotherapy, 2000. **46**(4): p. 293-302.

85. Rousey, S.R., et al., *Low-dose amphotericin B prophylaxis against invasive Aspergillus infections in allogeneic marrow transplantation.* Am J Med, 1991. **91**(5): p. 484-92.

86. Riley, D.K., et al., *The prophylactic use of low-dose amphotericin B in bone marrow transplant patients.* Am J Med, 1994. **97**(6): p. 509-14.

87. Perfect, J.R., et al., *Prophylactic intravenous amphotericin B in neutropenic autologous bone marrow transplant recipients.* J Infect Dis, 1992. **165**(5): p. 891-7.

88. Kelsey, S.M., et al., *Liposomal amphotericin (AmBisome) in the prophylaxis of fungal infections in neutropenic patients: a randomised, double-blind, placebo- controlled study.* Bone Marrow Transplant, 1999. **23**(2): p. 163-8.

89. Tollemar, J., et al., *Prophylactic use of liposomal amphotericin B (AmBisome) against fungal infections: a randomized trial in bone marrow transplant recipients.* Transplant Proc, 1993. **25**(1 Pt 2): p. 1495-7.

90. Tollemar, J., et al., *Randomized double-blind study of liposomal amphotericin B (Ambisome) prophylaxis of invasive fungal infections in bone marrow transplant recipients.* Bone Marrow Transplant, 1993. **12**(6): p. 577-82.

91. Penack, O., et al., *Low-dose liposomal amphotericin B in the prevention of invasive fungal infections in patients with prolonged neutropenia: results from a randomized, single-center trial.* Ann Oncol, 2006. **17**(8): p. 1306-12.

92. Cordonnier, C., et al., *Safety of a weekly high dose of liposomal amphotericin B for prophylaxis of invasive fungal infection in immunocompromised patients: PROPHYSOME Study.* Int J Antimicrob Agents, 2008. **31**(2): p. 135-41.

93. El-Cheikh, J., et al., *High-dose weekly liposomal amphotericin B antifungal prophylaxis following reduced-intensity conditioning allogeneic stem cell transplantation.* Bone Marrow Transplant, 2007. **39**(5): p. 301-6.

94. Annino, L., et al., *Prospective phase II single-center study of the safety of a single very high dose of liposomal amphotericin B for antifungal prophylaxis in patients with acute myeloid leukemia.* Antimicrob Agents Chemother, 2013. **57**(6): p. 2596-602.

95. Cornely, O.A., *Randomized comparison of liposomal amphotericin B versus placebo to prevent invasive mycoses in acute lymphoblastic leukemia.* J Antimicrob Chemother, , in press.

96. Rijnders, B.J., et al., *Aerosolized liposomal amphotericin B for the prevention of invasive pulmonary aspergillosis during prolonged neutropenia: a randomized, placebo-controlled trial.* Clin Infect Dis, 2008. **46**(9): p. 1401-8.

97. Alexander, B.D., et al., *Non-comparative evaluation of the safety of aerosolized amphotericin B lipid complex in patients undergoing allogeneic hematopoietic stem cell transplantation.* Transpl Infect Dis, 2006. **8**(1): p. 13-20.

98. Erjavec, Z., et al., *Tolerance and efficacy of Amphotericin B inhalations for prevention of invasive pulmonary aspergillosis in haematological patients.* Eur J Clin Microbiol Infect Dis, 1997. **16**(5): p. 364-8.

99. Schwartz, S., et al., *Aerosolized amphotericin B inhalations as prophylaxis of invasive aspergillus infections during prolonged neutropenia: results of a prospective randomized multicenter trial.* Blood, 1999. **93**(11): p. 3654-61.

100. Hertenstein, B., et al., *Low incidence of invasive fungal infections after bone marrow transplantation in patients receiving amphotericin B inhalations during neutropenia.* Ann Hematol, 1994. **68**(1): p. 21-6.

101. Hullard-Pulstinger, A., et al., *Prophylactic application of nebulized liposomal amphotericin B in hematologic patients with neutropenia.* Onkologie, 2011. **34**(5): p. 254-8.

102. Chong, G.L., et al., *Aerosolised liposomal amphotericin B to prevent aspergillosis in acute myeloid leukaemia: Efficacy and cost effectiveness in real-life.* Int J Antimicrob Agents, 2015. **46**(1): p. 82-7.

103. Cattaneo, C., et al., *A randomized comparison of caspofungin versus antifungal prophylaxis according to investigator policy in acute leukaemia patients undergoing induction chemotherapy (PROFIL-C study).* J Antimicrob Chemother, 2011. **66**(9): p. 2140-5.

104. Nachbaur, D., et al., *Primary antifungal prophylaxis with micafungin in patients with haematological malignancies: real-life data from a retrospective single-centre observational study.* Eur J Haematol, 2015. **94**(3): p. 258-64.
